# Ninjurin-1 mediates hepatic ischemia-reperfusion injury

**DOI:** 10.1126/sciadv.aeg9496

**Published:** 2026-08-26

**Authors:** Jan Mossemann, Brunna X. Martins, Yixuan Zhao, Fiorelle Aguilar, Daria Taskina, Claire J. Hur, Diana Nakib, Allen Volchuk, Gang Ye, Danish M. Ali, Ali Mirzaesmaeili, Iram Siddiqui, Ghazal Goodarzi, Philip J. Bilan, Irma B. Stowe, Nobuhiko Kayagaki, Sonya MacParland, Spencer A. Freeman, Neil M. Goldenberg, Benjamin E. Steinberg, Blayne A. Sayed

**Affiliations:** ^1^ Cell Biology Program, Hospital for Sick Children, Toronto, Canada.; ^2^ Department of General, Visceral, Thorax and Transplant Surgery, University Hospital of Cologne, Cologne, Germany.; ^3^ Neuroscience and Mental Health Program, Hospital for Sick Children, Toronto, Canada.; ^4^ Ajmera Transplant Centre, University Health Network, Toronto, Canada.; ^5^ Department of Paediatric Laboratory Medicine, Hospital for Sick Children, Toronto, Canada.; ^6^ Department of Physiological Chemistry, Genentech, South San Francisco, CA, USA.; ^7^ Department of Anesthesia and Pain Medicine, Hospital for Sick Children, Toronto, Canada.; ^8^ Division of General Surgery, Hospital for Sick Children, Toronto, Canada.

## Abstract

Lytic cell death pathways drive hepatic ischemia-reperfusion injury (IRI). The transmembrane protein ninjurin-1 (NINJ1) aggregates in the plasma membrane to permeabilize the cell during multiple cell death pathways implicated in hepatic IRI. We hypothesized that NINJ1 mediates liver IRI and that its inhibition would mitigate injury. We found that NINJ1 is highly expressed in human liver tissue and that its up-regulation and activation correlate with early allograft dysfunction in liver transplant patients. Using a segmental hepatic IRI model in mice and rats, *Ninj1* genetic deletion or pharmacologic inhibition diminished acute injury. Mice with hepatocyte- or macrophage-specific *Ninj1* knockout had reduced hepatocellular injury following IRI, suggesting that NINJ1 within both populations contributes to the resulting liver injury. Mechanistically, we found that hepatocytes and Kupffer cells are susceptible to hypoxia-induced NINJ1-mediated plasma membrane rupture, which can be pharmacologically prevented. We therefore position NINJ1 as a potential new therapeutic target to limit hepatic IRI, with important implications for liver transplantation.

## INTRODUCTION

Hepatic ischemia-reperfusion injury (IRI) is a two-hit phenomenon caused by temporarily interrupting liver perfusion (ischemia), followed by the re-establishment of normal blood flow (reperfusion). IRI occurs in a variety of settings, the most common being during liver transplantation and hemodynamic shock. Ischemia disrupts cellular homeostasis to activate both necrotic and regulated forms of cell death (*1*, *2*). Many of these dying cells lose structural integrity, thereby releasing cytoplasmic damage-associated molecular patterns (DAMPs), reactive oxygen species, and other inflammatory mediators into the local tissue environment. When blood flow is restored, the cytotoxic molecules released from dying cells spread throughout the tissue and into the systemic circulation, amplifying inflammation and secondary cellular stress and ultimately further propagating lytic cell death (*3*). Together, the local and systemic sequelae of hepatic IRI manifest as ischemia-reperfusion syndrome, a transient liver insufficiency and systemic sterile inflammatory response that drive acute patient morbidity and mortality (*3*, *4*). Severe acute IRI can also lead to long-term debilitating complications including liver fibrosis and, in the setting of transplant, predisposition to rejection (*5*).

Regulated lytic cell death pathways are important contributors to human liver disease (*6*–*8*), a finding recapitulated in animal models (*9*, *10*). The specific lytic cell death pathways activated in liver IRI include secondary necrosis, the breakdown of apoptotic bodies that are not removed by phagocytes at the site of injury (*11*), and pyroptosis, a proinflammatory death pathway that depends on the activation of pore-forming gasdermin proteins (*12*). Necroptosis and ferroptosis are other forms of lytic cell death implicated in hepatic IRI, especially in patients with comorbidities such as hepatic steatosis, advanced age, or iron overload (*13*–*16*). In each of secondary necrosis, pyroptosis and ferroptosis, the terminal event of plasma membrane rupture is dependent on the protein ninjurin-1 (NINJ1) (*17*–*19*). NINJ1 is a 16-kDa transmembrane protein with particularly high expression in the liver and innate immune cells (*20*, *21*). As NINJ1-expressing cells undergo cell death via these pathways, NINJ1 clusters into high-order oligomers within the plasma membrane to destabilize and disrupt the plasma membrane (*22*–*24*).

Early evidence with an anti-NINJ1 neutralizing antibody, termed clone D1, indicates that preventing NINJ1 clustering mitigates liver injury in multiple mouse models of acute injury, including IRI (*25*). As compared to isotype control, clone D1 administration 4 hours prior to hepatic IRI reduced circulating markers of hepatocellular injury and liver cell death (*25*). These findings are consistent with the known role of plasma membrane rupture in the multiple cell death pathways that propagate liver damage in IRI. Similarly, the cytoprotective amino acid glycine and small molecule muscimol preserve cellular integrity by inhibiting NINJ1 activation (*26*, *27*), positioning NINJ1 as a viable therapeutic target. We therefore hypothesized that NINJ1 activation occurs in human IRI and that its genetic deletion or pharmacologic inhibition would protect the liver from injury in animal models. Here, we demonstrate that NINJ1 is expressed in human liver and that human liver allografts with severe early allograft dysfunction posttransplant have increased activation of NINJ1. In both mouse and rat *Ninj1* knockout (KO) models, the absence of NINJ1 diminishes injury from IRI, substantiating its role in driving hepatocellular damage. Compartmental NINJ1 deletion in hepatocytes or macrophages confer protection against acute IRI, establishing a role for NINJ1 in both cell types in driving IRI, a finding that we substantiate in an in vitro hypoxia-reoxygenation model. Together, our findings demonstrate a crucial role of NINJ1 in mediating acute hepatic IRI and indicate that targeting NINJ1 as a therapeutic target may dampen the severity of hepatic IRI.

## RESULTS

### NINJ1 is expressed in the human liver

**Fig. 1.**
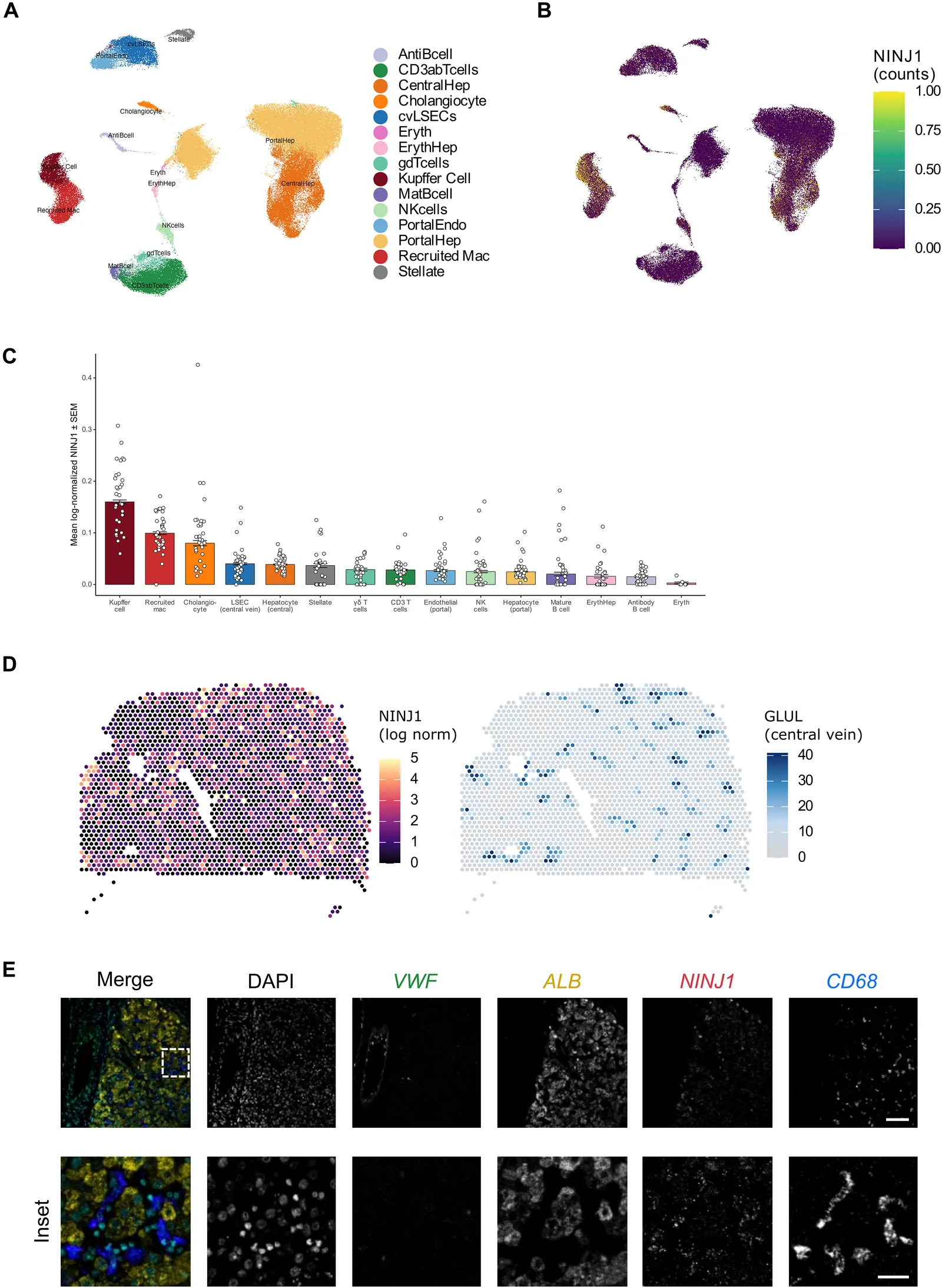
NINJ1 is expressed across cell types in the healthy human liver. (**A**) UMAP of 113,544 single cells from 24 NDD healthy human liver samples (*28*). Cells are colored by annotated cell type. (**B**) UMAP as in (A), colored by *NINJ1* gene expression counts. (**C**) Mean *NINJ1* expression per cell type across all 15 annotated populations, ordered from highest to lowest. Bars represent mean log-normalized expression ± SEM. Individual NDD liver samples are shown as circles. NK cells, natural killer cells. (**D**) Visium spatial transcriptomic expression of *NINJ1* in a representative NDD liver tissue section (left) and GLUL expression representing central venous hepatocytes (right). Spearman correlation between NINJ1 and GLUL across all spots: ρ = 0.21, *P* < 0.0001, *n* = 2277 spots. (**E**) In situ hybridization using RNAscope of human liver shows coexpression of *NINJ1* (red) with markers of hepatocytes (*ALB*; yellow) and macrophages (*CD68*; blue). Endothelial cells were identified by expression of *VWF* (green). Nuclei stained with DAPI (cyan in merged image). Scale bars, 100 μm (top row) and 30 μm for the inset (bottom row).

Genome-wide association studies have identified a human *NINJ1* single-nucleotide polymorphism (rs7018885), which is associated with decreased liver transaminase levels (*25*); however, the role of NINJ1 in human liver transplantation has not been previously explored. Using a publicly available single-cell RNA-sequencing (scRNA-seq) dataset from 24 human neurologically deceased donor (NDD) hepatic allografts used for transplantation comprising 113,544 single cells (*28*), we examined NINJ1 transcript expression and distribution across immune and parenchymal clusters on Uniform Manifold Approximation and Projection (UMAP) embeddings (Fig. 1, A and B). *NINJ1* expression was detected across all annotated cell types, with the greatest enrichment within myeloid populations, most prominent in Kupffer cells, as well as in parenchymal hepatocyte populations (Fig. 1, B and C). Quantitative analysis confirmed that Kupffer cells express NINJ1 at significantly higher levels than hepatocytes, with both a greater mean expression and a higher proportion of NINJ1-expressing cells in the Kupffer cell population as compared with either central or portal hepatocytes (fig. S1). Liver sinusoidal endothelial cells (LSECs) expressed *NINJ1* at levels comparable to hepatocytes, approximately fivefold lower than Kupffer cells (figs. S1B and S3).

We next interrogated nondissociative Visium spatial transcriptomic data from intact NDD liver sections, which confirmed *NINJ1* expression in situ but did not reveal a pronounced lobular zonation pattern, with only a weak correlation between NINJ1 and GLUL expression, a marker of central venous hepatocytes (Spearman ρ = 0.21, *P* < 0.0001; Fig. 1D). We corroborated the spatial transcriptomics data using RNAscope in situ hybridization to examine the expression of *NINJ1* in healthy human liver samples. *NINJ1* mRNA was expressed across cell types and was particularly prominent in the parenchyma as compared to portal tracts (Fig. 1E). *NINJ1* expression strongly colocalized with *CD68*, a macrophage and Kupffer cell marker, which were well visualized in the sinusoidal spaces (Fig. 1E). *NINJ1* colocalized to a lesser extent with albumin (*ALB*)–expressing hepatocytes (Fig. 1E).

### Hepatic IRI activates NINJ1 in human liver transplant

**Fig. 2.**
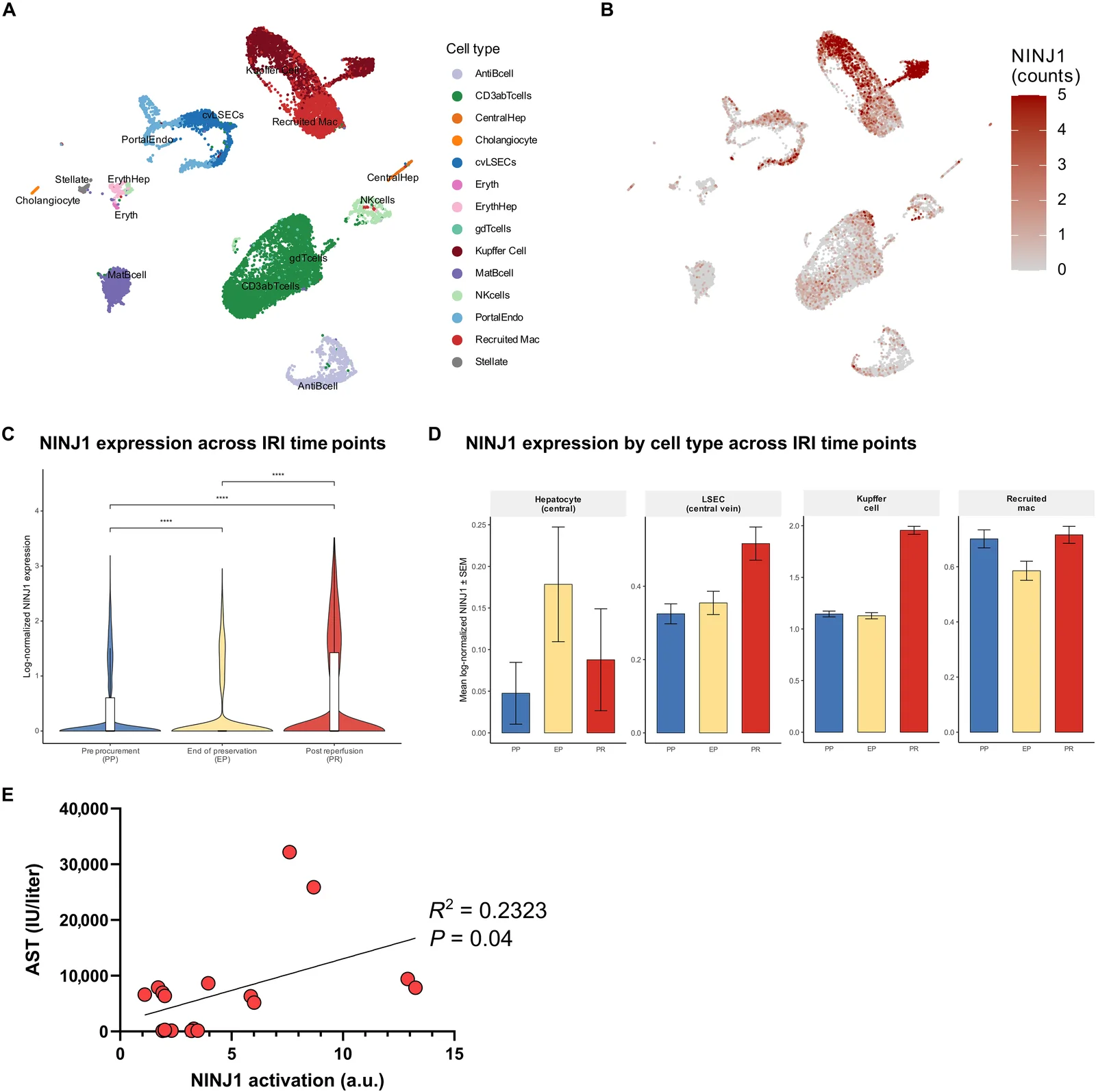
NINJ1 expression is up-regulated in human liver and oligomerizes in ischemia-reperfusion. (**A**) UMAP plot of 14,311 single cells from human liver samples across three time points of a liver transplant procedure: PP, EP, and 2 hours PR (*29*), with cell annotations transferred from (*28*). (**B**) UMAP colored by *NINJ1* gene expression. (**C**) Violin plots showing log-normalized *NINJ1* expression across the three IRI time points (PP, EP, and PR). Center line represents the median; box represents interquartile range. Statistical comparisons performed by Wilcoxon rank-sum test; PP versus EP: *P* = 4.18 × 10^−5^; PP versus PR: *P* = 1.11 × 10^−42^; EP versus PR: *P* = 7.09 × 10^−64^. (**D**) Mean log-normalized *NINJ1* expression ±SEM per time point in hepatocytes, LSECs, Kupffer cells, and recruited macrophages. (**E**) PR AST levels following human liver transplant in 18 samples as a function of NINJ1 activation in the liver graft tissue. The extent of NINJ1 activation was assayed using blue native–PAGE on liver graft tissue. Linear regression demonstrates the positive relationship between AST levels (surrogate measure of liver injury) and NINJ1 activation (*R*^2^ = 0.2323; *P* = 0.04). a.u., arbitrary unit.

Given the expression of *NINJ1* in both Kupffer cell and hepatocyte populations, we next sought to interrogate *NINJ1* expression dynamics in the setting of human hepatic IRI. We analyzed a publicly available scRNA-seq dataset comprising human liver samples collected at three time points during liver transplantation: preprocurement (PP; baseline prior to cold ischemia), end of preservation (EP; following cold ischemia), and 2 hours postreperfusion (PR) (*29*). Cell-type annotations were assigned by label transfer from our healthy donor liver reference atlas (*28*). *NINJ1* expression was detected across all major hepatic cell populations at each time point (Fig. 2, A and B, and fig. S2A). Compared with the PP baseline, NINJ1 transcript levels were significantly elevated following reperfusion across all cells (PP versus EP: *P* = 4.18 × 10^–5^, PP versus PR: *P* = 1.11 × 10^–42^, EP versus PR: *P* = 7.09 × 10^–64^; Fig. 2C). Cell-type stratified analysis demonstrated that this PR increase in NINJ1 expression was observed across hepatocytes, LSECs, Kupffer cells, and recruited macrophages (Fig. 2D), consistent with the broad cellular expression of *NINJ1* identified in the healthy liver and supporting a role for NINJ1 across multiple hepatic cell compartments during IRI.

To investigate whether *NINJ1* expression in Kupffer cells is associated with broader injury-related transcriptional programs, we performed a Spearman correlation analysis between *NINJ1* and all expressed genes within the Kupffer cell population across the three IRI time points (fig. S2, B and C). At PR, *NINJ1* expression was significantly coexpressed with genes characteristic of inflammatory Kupffer cell activation, including *ICAM1* (ρ = 0.35), *CCL4* (ρ = 0.31), *NFKB1* (ρ = 0.24), *IL1RN* (ρ = 0.25), *SOD2* (ρ = 0.25), and multiple tumor necrosis factor–response genes (*TNFAIP2*, *TNFAIP8*, and *TNIP3*). This coexpression pattern was substantially weaker at PP, indicating that the association between *NINJ1* and inflammatory gene programs emerges specifically in the context of reperfusion injury. Together, these data suggest that *NINJ1* up-regulation in Kupffer cells PR is coupled to a broader nuclear factor κB–driven inflammatory response, consistent with a role for NINJ1 in injury-activated macrophage biology.

Having established increased *NINJ1* expression post-IRI, we aimed to determine whether NINJ1 was activated in the liver following IRI. We examined transplanted human hepatic allografts (table S1) and characterized posttransplant graft injury based on serum markers of liver damage in the recipients based on serum aspartate aminotransferase (AST) levels (*30*). Cryopreserved samples of human liver allografts PR (prior to abdominal closure during primary transplant) were analyzed for NINJ1 activation by blue native–polyacrylamide gel electrophoresis (PAGE) (Fig. 2E and fig. S4). With this approach, which maintains native protein complexes, activated and polymerized NINJ1 shifts to a series of high–molecular weight bands (*17*, *26*). We looked for an association between AST levels within 2 days posttransplant and NINJ1 activation (Fig. 2E), which demonstrated a positive relationship between NINJ1 activation and IRI severity (*R*^2^ = 0.2323; *P* = 0.04). These data are consistent with enhanced NINJ1 clustering and activation in injured human liver allografts following transplantation.

### Hepatic IRI activates NINJ1 in a mouse model

**Fig. 3.**
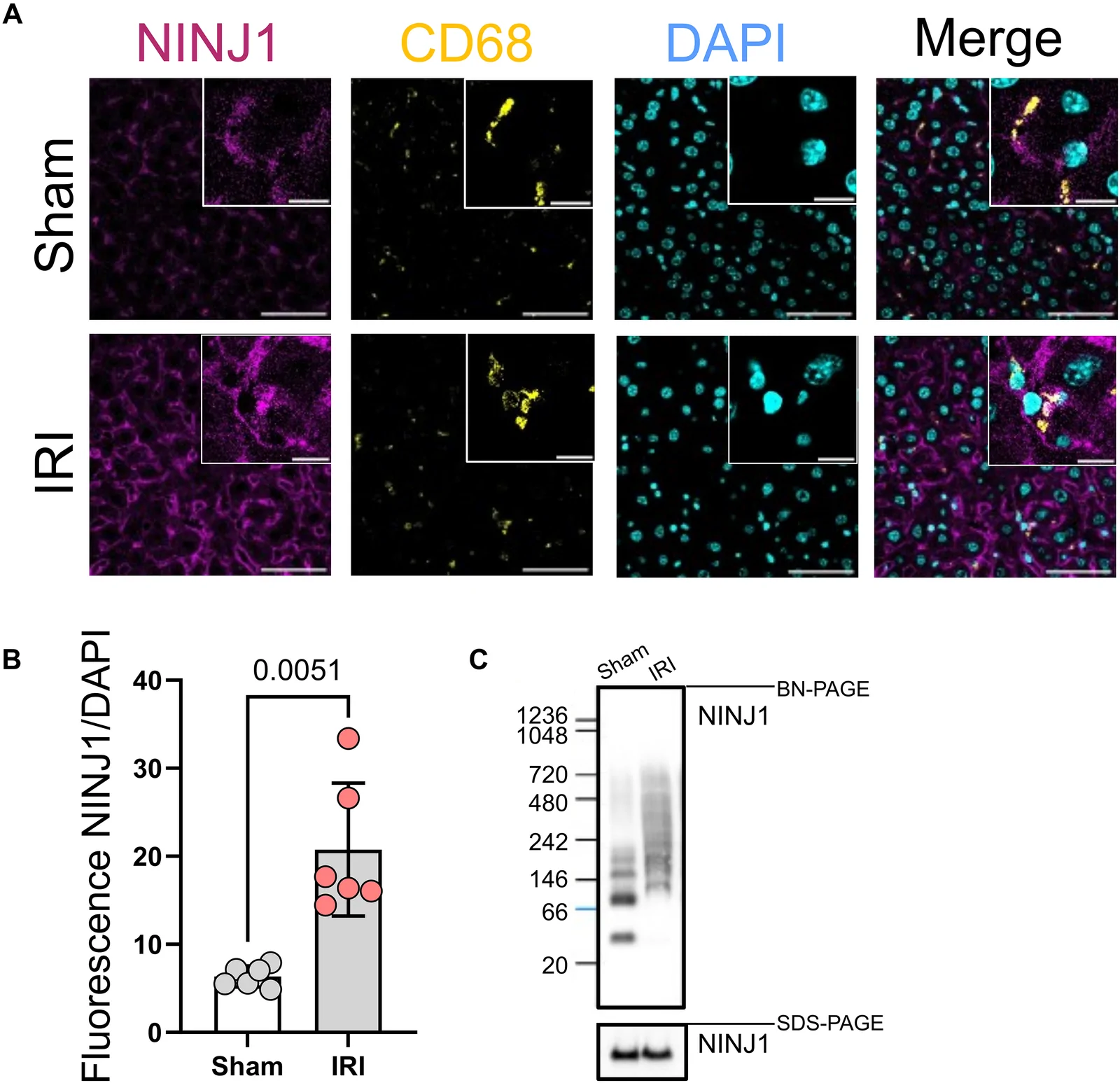
NINJ1 increases and oligomerizes following IRI in a mouse model. Mixed-sex cohorts of WT mice underwent IRI consisting of 1 hour of 70% segmental warm ischemia followed by 6 hours of reperfusion prior to tissue collection. Sham laparotomy was used as a negative control. (**A**) Representative immunofluorescence of NINJ1 (magenta) in liver tissue from sham-operated or IRI liver. Kupffer cells were identified by CD68 (yellow). Scale bars, 50 and 10 μm for the full fields of view and insets, respectively. (**B**) Quantification of total NINJ1 fluorescence in liver tissue from sham-operated (*N* = 6) and IRI (*N* = 6) animals as normalized to DAPI. *P* value was determined by T test. (**C**) Representative blue native (BN)–PAGE for NINJ1 in liver lysates from sham-operated and IRI-treated animals. SDS-PAGE for NINJ1 provided as a loading control.

Having established that *NINJ1* is expressed in the human liver and that its activation correlates with heightened early allograft dysfunction following transplant, we turned to a mouse model to further delineate the mechanistic role of NINJ1 in IRI. In our model, mice were subjected to 70% segmental liver ischemia for 1 hour followed by 6 hours of reperfusion and compared to a sham laparotomy. In sham-operated animals, NINJ1 colocalized with both hepatocytes and Kupffer cells with NINJ1 expression enhancing during IRI (Fig. 3, A and B, and fig. S5). Blue native–PAGE of liver lysates from sham-treated mice showed that NINJ1 migrates as distinct dimers, tetramers, and low-order oligomers (Fig. 3C), consistent with the proposed resting oligomer state of NINJ1 (*31*). In contrast, IRI induced oligomerization of NINJ1 into higher molecular weight complexes, while diminishing the abundance of NINJ1 dimers (Fig. 3C). Together, these data demonstrate that NINJ1 is expressed in hepatocyte and Kupffer cell populations and establish that NINJ1 is activated and has increased hepatocyte abundance during mouse IRI.

### *Ninj1* KO limits mouse and rat hepatic IRI

**Fig. 4.**
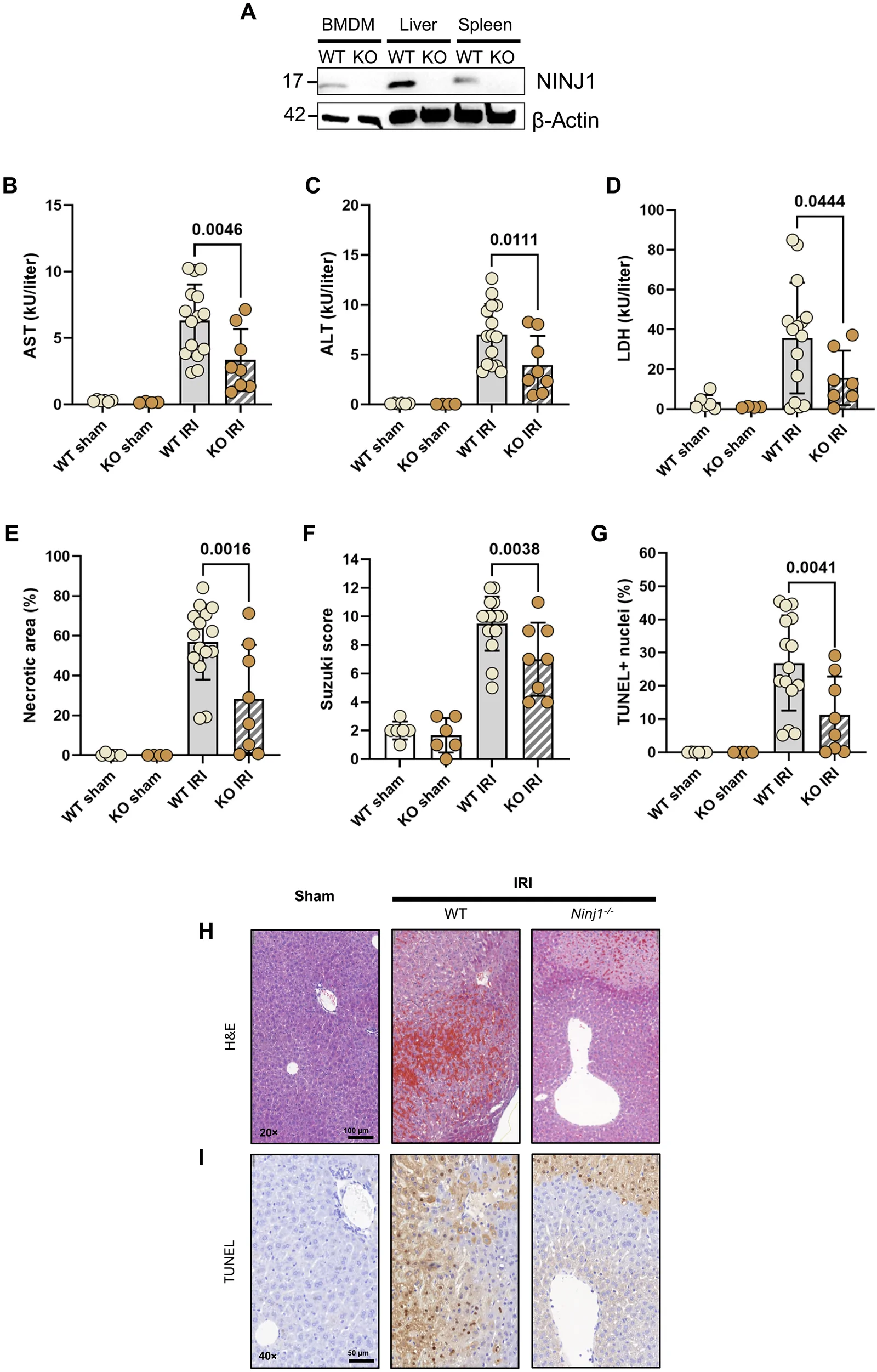
*Ninj1* KO protects against liver IRI in mice. Mixed-sex cohorts of *Ninj1* KO mice or their littermate controls underwent IRI consisting of 1 hour of 70% segmental warm ischemia followed by 6 hours of reperfusion prior to tissue collection. Sham laparotomy was used as a negative control. Group sizes: sham WT, *N* = 6; sham KO, *N* = 4; IRI WT, *N* = 15; and IRI KO, *N* = 8. (**A**) SDS-PAGE showing NINJ1 expression in mouse BMDM, liver, and spleen homogenates from WT animals compared with *Ninj1* KO animals. (**B** to **D**) Hepatocyte-specific injury was assayed by serum AST and ALT, and general cellular cytotoxicity was measured by serum LDH. Note one missing data point in the KO IRI group for the LDH assay due to inadequate sample for analysis. Data points represent individual animals with the mean and standard error of the mean superimposed. *P* values were determined by analysis of variance (ANOVA) with Tukey’s multiple comparison correction. (**E** to **I**) *Ninj1* KO mice exposed to IRI had decreased liver tissue necrosis (E), Suzuki score (F), and cell death as assayed by TUNEL staining (G) compared to their littermate controls. Representative images of hematoxylin and eosin (H&E) (H) and TUNEL staining (I) are shown. Group sizes and statistical analysis are the same as described for (B) to (D), above. Images in (H) and (I) are reproduced in fig. S6B to demonstrate how confluent necrosis and TUNEL-positive nuclei were classified in an automated fashion. Data points represent individual animals with the mean and standard error of the mean superimposed. *P* values were determined by ANOVA with Šidák’s multiple comparison correction.

To determine whether loss of NINJ1 offers protection from hepatic IRI, wild-type (WT) and whole-animal *Ninj1* KO mice (*Ninj1^−/−^*) (Fig. 4A) (*17*) were subjected to acute IRI. Serum lactate dehydrogenase (LDH; an indicator of cell rupture and nonspecific tissue injury) and aminotransferases [AST and alanine aminotransferase (ALT); serum markers of hepatocyte injury] were used to evaluate the extent of liver injury (Fig. 4, B to D). Both WT and *Ninj1^−/−^* sham-operated mice did not show appreciable biochemical evidence of hepatocellular injury in their serum (Fig. 4, B to D). Hepatic IRI induced marked evidence of hepatocellular injury in WT mice, whereas *Ninj1^−/−^* mice had ∼50% less biochemical evidence of liver injury following IRI compared with their WT counterparts (Fig. 4, B to D). Using the Suzuki scoring scale of histopathologic liver injury for IRI, the extent of liver injury was reduced in *Ninj1^−/−^* mice as compared to WT controls (Fig. 4F) (*32*, *33*), including a reduction of confluent necrosis (WT = 56.9 ± 4.9, *N* = 15; KO = 28.3 ± 9.6, *N* = 8; *P* = 0.0016; Fig. 4, E and H, and fig. S6). Last, cell death was quantified using the TUNEL (terminal deoxynucleotidyl transferase–mediated deoxyuridine triphosphate nick end labeling) assay, which stains fragmented DNA to identify cells undergoing cell death (Fig. 4, G and I, and fig. S6) (*34*). Nearly one third of nuclei were TUNEL-positive in livers from WT mice injured by IRI, compared with 12% TUNEL-positive nuclei in the *Ninj1*^−/−^ animals (Fig. 4G). Notably, early inflammatory cell infiltration into the liver was not different between injured *Ninj1* WT and KO mice (fig. S6). Minimal neutrophils, quantified as Ly6G-positive cells by immunohistochemistry, were observed in the livers of sham animals. In IRI mice, there was a comparable increase in neutrophil infiltration between genotypes (fig. S7A). F4/80-positive macrophages resident in the liver did not change significantly in either genotype following IRI, although there was a trend toward fewer macrophages (fig. S7B) consistent with an observed early depletion of Kupffer cells in IRI (*35*).

**Fig. 5.**
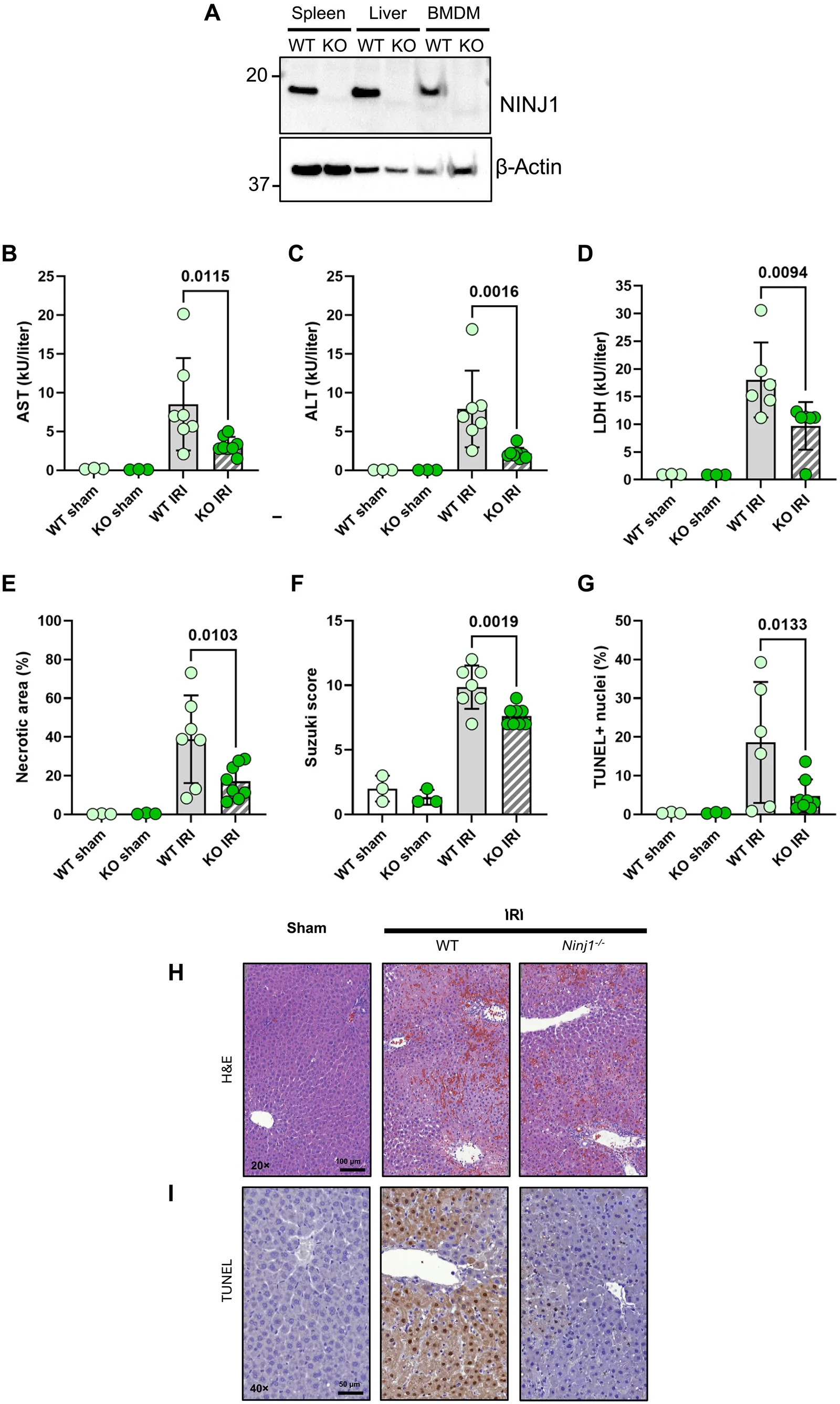
*Ninj1* KO protects against liver IRI in rats. Mixed-sex cohorts of *Ninj1* KO rats or their littermate controls underwent IRI consisting of 1 hour of 70% segmental warm ischemia followed by 6 hours of reperfusion prior to tissue collection. Sham laparotomy was used as a negative control. Group sizes: sham WT, *N* = 3; sham KO, *N* = 3; IRI WT, *N* = 7; and IRI KO, *N* = 8. (**A**) SDS-PAGE demonstrating NINJ1 expression in rat BMDMs, liver, and spleen homogenates from WT animals compared with *Ninj1* KO animals. (**B** to **D**) Hepatocyte-specific injury was assayed by serum AST and ALT, and general cellular cytotoxicity was measured by serum LDH. Data points represent individual animals with the mean and standard error of the mean superimposed. *P* values were determined by ANOVA with Tukey’s multiple comparison correction. (**E** to **I**) *Ninj1* KO rats exposed to IRI had decreased necrosis (E), Suzuki score (F), and cell death as assayed by TUNEL staining (G) compared to WT controls. Representative liver histology images of H&E (H) and TUNEL staining (I) are shown. Group sizes and statistical analysis are the same as described for (B) to (D), above. Data points represent individual animals with the mean and standard error of the mean superimposed. *P* values were determined by ANOVA with Šidák’s multiple comparison correction.

We next evaluated whether the function of NINJ1 in IRI is conserved across species using *Ninj1^−/−^* KO rats (Fig. 5A). This interspecies comparison is translationally important given the known differences between mice and rats in factors that can affect IRI, including metabolic pathways (e.g., CYP450 system), toxin sensitivity, gene expression profiles, and immune responses between mice and rats (*36*, *37*). *Ninj1^−/−^* rats had no evident basal phenotype, displaying normal home cage behavior, and growth (fig. S8A). To first determine whether NINJ1 control of plasma membrane rupture is conserved in rats, bone marrow–derived macrophages (BMDMs) were cultured from *Ninj1^−/−^* and WT rats. Pyroptosis [lipopolysaccharide (LPS) and nigericin] and secondary necrosis (ABT-199 and doxorubicin) were induced, and plasma membrane rupture was evaluated by LDH release into the supernatant. LDH release from *Ninj1^−/−^* BMDMs was significantly abrogated as compared to WT-derived cells, confirming NINJ1-dependent control of plasma membrane rupture in rat macrophages (fig. S8, B and C).

The *Ninj1^−/−^* rats and their WT counterparts were subjected to a similar segmental model of ischemia (1 hour) and reperfusion (6 hours) as our mouse cohorts. *Ninj1^−/−^* rats had significant protection from hepatic IRI, as demonstrated by a reduction in AST, ALT, and LDH compared to WT controls exposed to IRI (Fig. 5, B to D). Histopathologic analysis of the liver using the Suzuki score revealed decreased levels of necrosis (Fig. 5E), congestion, and vacuolization in injured *Ninj1^−/−^* rats compared to WT (Fig. 5, E, F, and H). In addition, cell death scored by TUNEL-positive nuclei revealed a marked decrease in injured *Ninj1 ^−/−^* rats compared to WT (Fig. 5, G and I). These data in both mouse and rat indicate that NINJ1 deletion protects the liver against IRI across species.

### Pharmacologic NINJ1 inhibition with glycine reduces NINJ1 activation and hepatic IRI in a mouse model

**Fig. 6.**
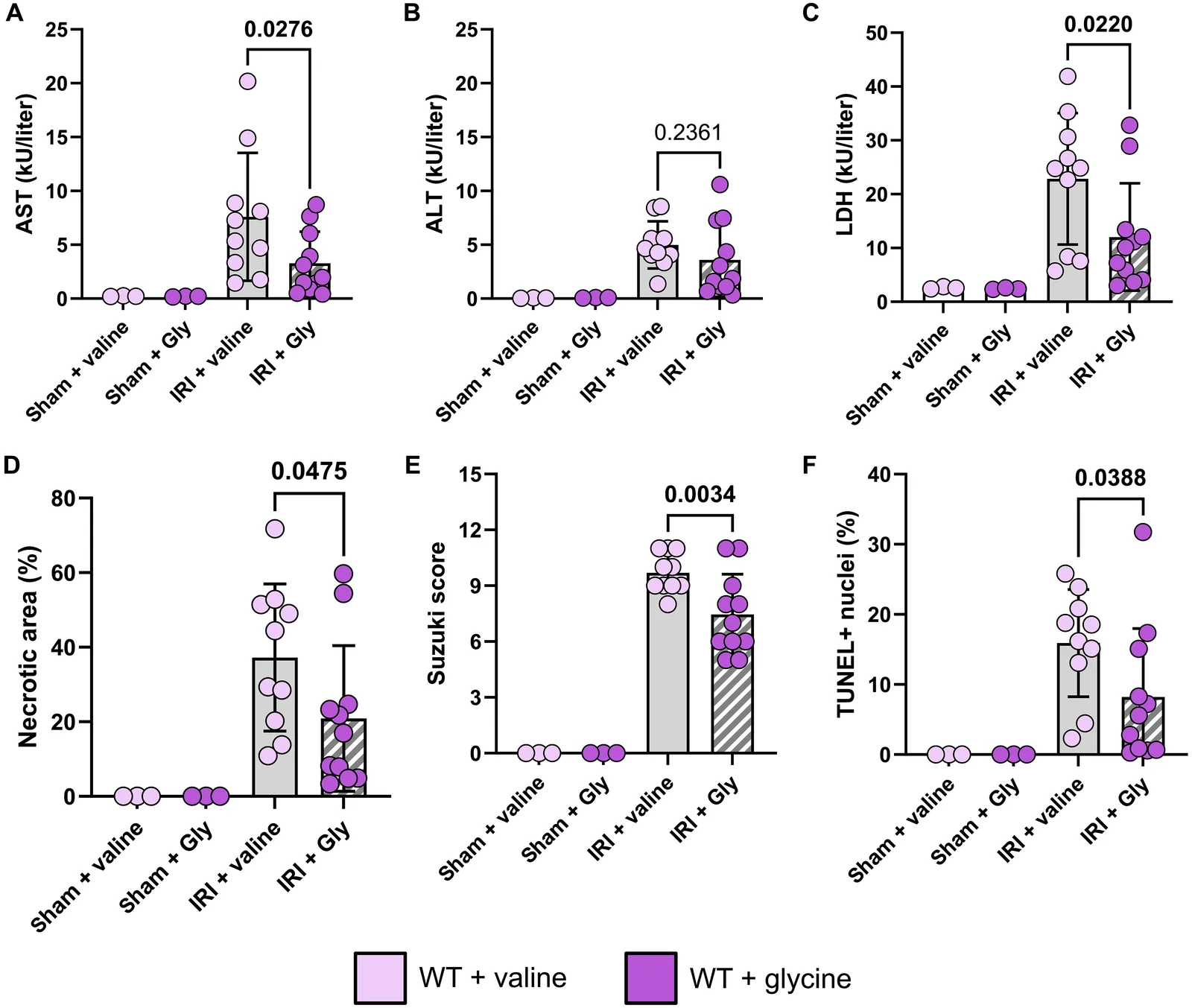
Glycine administration protects against liver IRI. WT C57BL/6 mice were administered with 0.5 mg/kg of sterile glycine (Gly) or valine in PBS intraperitoneal 1 hour prior to ischemia and 0.5 mg/kg glycine or valine immediately following ischemia, followed by 6 hours of reperfusion. Group sizes: sham valine, *N* = 3; sham glycine, *N* = 3; IRI valine, *N* = 10; and IRI glycine, *N* = 11. (**A** to **C**) Hepatocyte-specific injury was assayed by serum AST and ALT, and general cellular cytotoxicity was measured by serum LDH. (**D** to **F**) Liver tissue analysis of the percent necrotic area, Suzuki score, and the percentage of TUNEL-positive nuclei in the total area of the left lateral lobe and the left and right median lobes of the liver. Data points represent individual animals with the mean and standard error of the mean superimposed. *P* values were determined by ANOVA with Šidák’s multiple comparison correction.

Empiric evidence supports the role of the amino acid glycine as a cytoprotective agent in organ preservation during IRI and transplant (*38*–*43*). We previously demonstrated that glycine cytoprotection works at the level of NINJ1, inhibiting its oligomerization and thus activation within the plasma membrane (*26*). To determine whether glycine protection from hepatic IRI correlates with NINJ1 inhibition, WT C57BL/6 mice were administered glycine by intraperitoneal injection 1 hour prior to the induction of hepatic ischemia and directly following the beginning of reperfusion. An equal dose of valine, an amino acid without cytoprotective properties (*26*, *44*), was administered to WT mice as a placebo control. Glycine treatment also significantly lowered serum levels of AST, ALT, and LDH induced by IRI compared to control mice (Fig. 6, A to C) and reduced confluent necrosis and percent TUNEL-positive nuclei relative to valine-treated animas (Fig. 6, D to F). Thus, pharmacologic inhibition of NINJ1 activation protects against liver injury following ischemia and reperfusion.

### Both macrophage and hepatocyte NINJ1 contribute to mouse IRI

**Fig. 7.**
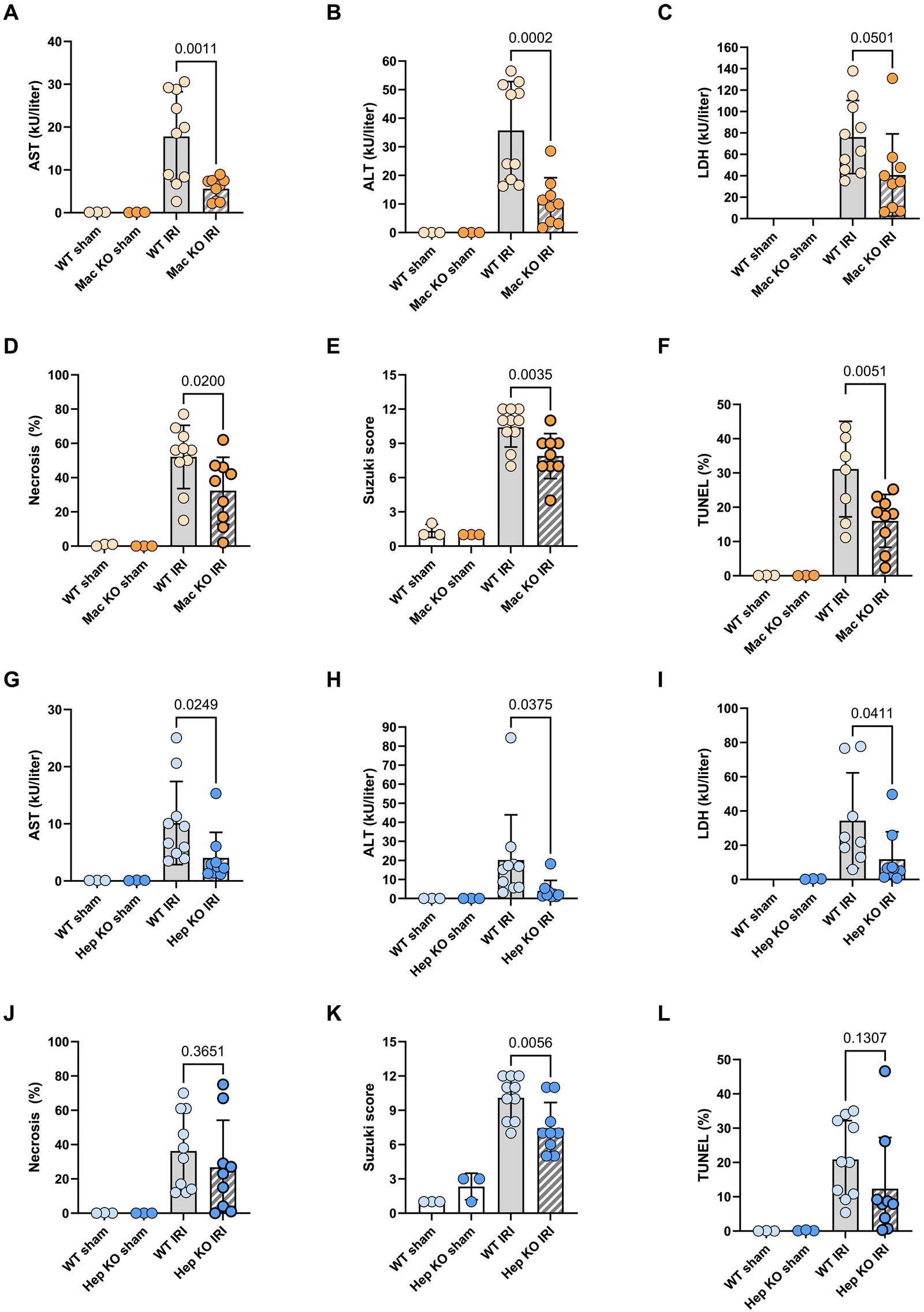
Macrophage-specific and hepatocyte-specific *Ninj1* KO protects against liver IRI in mice. (**A** to **F**) Mixed-sex cohorts of macrophage–specific *Ninj1* KO mice or their littermate controls underwent IRI consisting of 1 hour of 70% segmental warm ischemia followed by 6 hours of reperfusion prior to tissue collection. Sham laparotomy was used as a negative control. Group sizes: sham WT, *N* = 3; sham macrophage KO, *N* = 3; IRI WT, *N* = 10; and IRI macrophage KO, *N* = 9. (A to C) Hepatocyte-specific injury was assayed by serum AST and ALT, and general cellular cytotoxicity was measured by serum LDH. LDH data for sham-operated animals are unavailable due to inadequate sample for analysis. (D to F) Liver tissue analysis of the percent necrotic area, Suzuki score, and the percentage of TUNEL-positive nuclei in the total area of the left lateral lobe and the left and right median lobes of the liver. (**G** to **L**) Mixed-sex cohorts of hepatocyte (Hep)–specific *Ninj1* KO mice or their littermate controls underwent IRI consisting of 1 hour of 70% segmental warm ischemia followed by 6 hours of reperfusion prior to tissue collection. Sham laparotomy was used as a negative control. Group sizes: sham WT, *N* = 3; sham hepatocyte KO, *N* = 3; IRI WT, *N* = 10; and IRI hepatocyte KO, *N* = 9. (G to I) Hepatocyte-specific injury was assayed by serum AST and ALT, and general cellular cytotoxicity was measured by serum LDH. LDH data for WT sham-operated animals are unavailable due to inadequate sample for analysis. (J to L) Liver tissue analysis of the percent necrotic area, Suzuki score, and the percentage of TUNEL-positive nuclei in the total area of the left lateral lobe and the left and right median lobes of the liver. Data points represent individual animals with the mean and standard error of the mean superimposed. *P* values were determined by ANOVA with Šidák’s multiple comparison correction.

Our data show that both Kupffer cells and hepatocytes express NINJ1 (Figs. 1 and 2 and fig. S1). Kupffer cells, the primary resident hepatic macrophages, make up 15 to 20% of normal murine livers (*45*) and are known to mediate hepatic IRI (*46*, *47*). While hepatocytes make up 50 to 70% of the cells in the liver (*45*), the role of hepatocyte rupture in propagating IRI remains undefined. Our genetic knockout (Figs. 4 and 5) and pharmacologic (Fig. 6) approaches do not distinguish in which cell populations NINJ1 activation contributes to IRI. To delineate which NINJ1-expressing cell type(s) drive our observed protective phenotype in the whole animal KO models (Figs. 4 and 5), we used a conditional *Ninj1* KO mouse (*25*). We targeted NINJ1 in the Kupffer cell and hepatocyte populations using *Ninj1* floxed mice crossed with either LysM-cre or Alb-cre, respectively, which were compared to littermate WT controls (*Ninj1* flox^+/+^ Cre^−/−^). Specific deletion of *Ninj1* in either macrophages or hepatocytes decreased serum markers (Fig. 7, A to C and G to I) and histological evidence of IRI (Fig. 7, D to F and J to L). Of note, hepatocyte-specific *Ninj1* deletion did not limit the extent of confluent necrosis or TUNEL-positive cells following IRI (Fig. 7, J and L).

### Primary murine Kupffer cells and hepatocytes undergo NINJ1-mediated plasma membrane rupture in an in vitro hypoxia-reoxygenation model

**Fig. 8.**
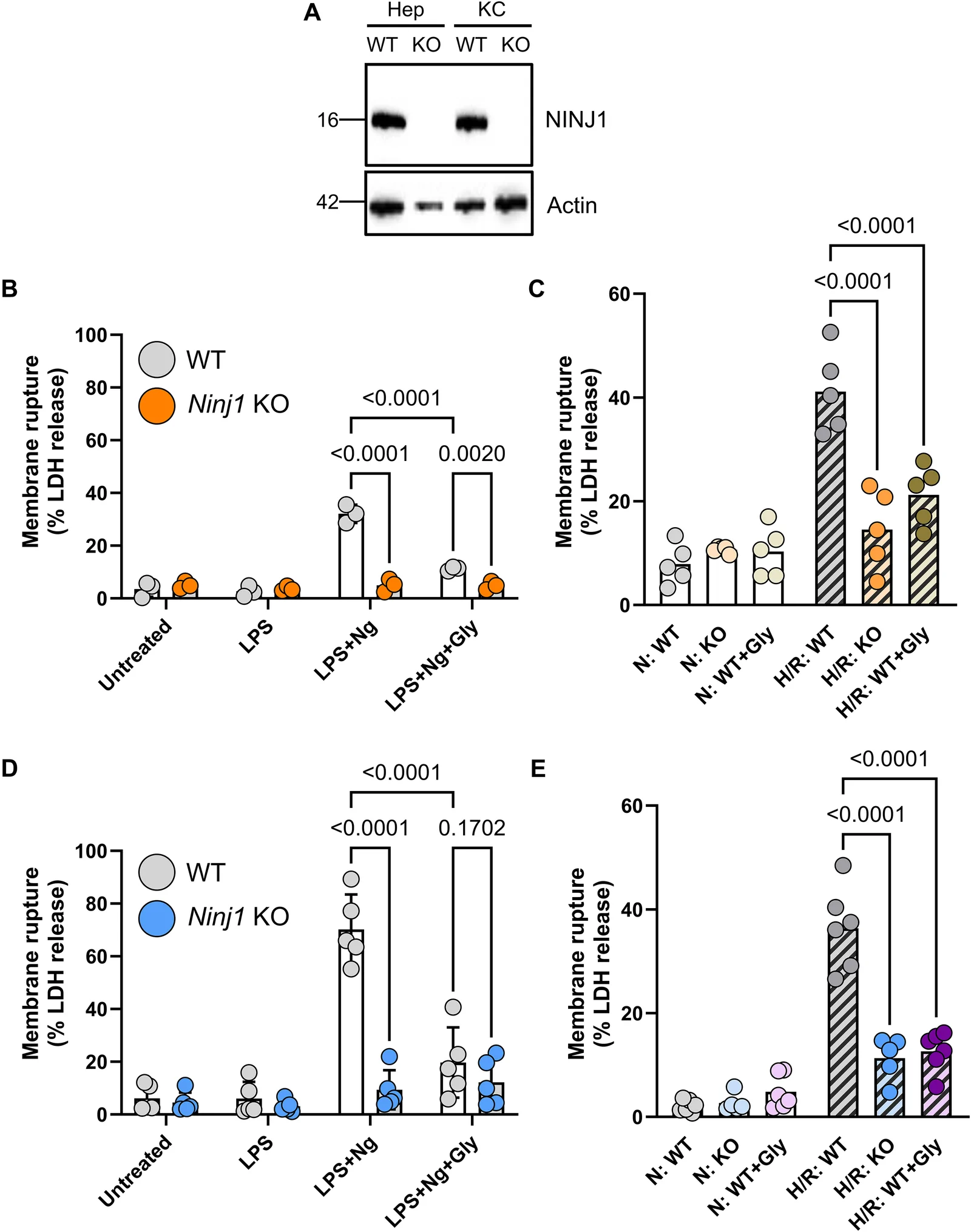
NINJ1 mediates plasma membrane rupture in hepatocytes and macrophages. (**A**) Primary hepatocytes and Kupffer cells (KC) were purified from WT and *Ninj1* KO mice. NINJ1 expression was confirmed by Western blot. β-Actin is shown as a protein loading control. (**B** to **E**) Pyroptosis was induced by priming with LPS (3 hours) followed by treatment with nigericin (Ng) (2 hours). Untreated and LPS-alone are shown as controls. Hypoxia-reoxygenation injury was induced by incubating the cells in 1% O_2_ for 1 hour followed by normoxic conditions (21% O_2_) for 5 hours. Normoxia for 6 hours served as the control. Where indicated, cells were cotreated with glycine (5 mM). Membrane rupture was measured by LDH release. (B and C) Kupffer cells underwent (B) pyroptosis and (C) hypoxia-reoxygenation injury. In both models, glycine (5 mM) significantly reduced LDH release. *Ninj1* KO Kupffer cells were protected from hypoxia-induced membrane rupture. (D and E) Hepatocytes underwent (D) pyroptosis and (E) hypoxia-reoxygenation injury. Glycine treatment (5 mM) decreased LDH release in WT cells in both contexts, while *Ninj1* KO hepatocytes showed resistance to membrane rupture under hypoxia-reoxygenation. Data points represent independent experiments from cells derived from different animals with the mean and standard error of the mean superimposed. *P* values were determined by ANOVA with Šidák’s multiple comparison correction. N, normoxia; H/R, hypoxia-reoxygenation.

Our in vivo data implicate both hepatocytes and Kupffer NINJ1 in hepatic IRI. To determine whether Kupffer cells are susceptible to NINJ1-mediated lytic cell death, primary Kupffer cells were isolated from the livers of *Ninj1^−/−^* and WT mice (Fig. 8A). We confirmed that these cells were protected from plasma membrane rupture during pyroptosis when genetically devoid of *Ninj1*. Pyroptosis was induced by priming the cells with LPS followed by treatment with the NLRP3 inflammasome-activating ionophore nigericin. Pyroptosis induction led to membrane rupture as quantified by LDH release (Fig. 8B). LPS alone did not induce cell rupture, whereas LPS plus nigericin induced cell rupture in WT Kupffer cells, which was significantly decreased in *Ninj1^−/−^* Kupffer cells (Fig. 8B). The magnitude of this effect was comparable to the pretreatment of WT Kupffer cells with the cytoprotective agent glycine. Glycine did not confer additional protection to *Ninj1* KO Kupffer cells (Fig. 8B), consistent with the concept that glycine is acting at the level of NINJ1 (*26*).

To further delineate the mechanistic basis of NINJ1 activity in the Kupffer cell during IRI, we turned to an in vitro model of IRI consisting of hypoxic culture (1 hour at 1% O_2_) followed by normoxia for 5 hours (*48*). Hypoxia-reoxygenation induced lytic cell death in WT Kupffer cells, which was significantly reduced in *Ninj1^−/−^* cells (Fig. 8C). Glycine treatment protected WT Kupffer cells from pyroptosis and hypoxia-reoxygenation but did not confer any additional protection to *Ninj1* KO Kupffer cells (Fig. 8, B and C). Although glycine-treated *Ninj1* KO Kupffer cells release less LDH than glycine-treated WT Kupffer cells, LDH release is unchanged between KO Kupffer cells with or without glycine. These findings are consistent with the notion that glycine mediates its cytoprotective effects at the level of NINJ1 activation (*26*).

To determine whether hepatocytes can similarly undergo active NINJ1-mediated plasma membrane rupture in vitro, primary hepatocytes were isolated from *Ninj1^−/−^* and WT littermates (Fig. 8A). The cells were cultured in the presence of no stimulation, LPS alone, LPS plus nigericin, or LPS plus nigericin and glycine. LPS alone did not induce lytic cell death, but LPS with nigericin induced pyroptosis in ∼70% of cells (Fig. 8D). Hepatocytes isolated from *Ninj1^−/−^* mice were resistant to plasma membrane rupture and glycine pretreatment decreased LDH release from WT hepatocytes (Fig. 8D). Glycine treatment of WT hepatocytes had a comparable effect to *Ninj1^−/−^* cells (Fig. 8D). A similar phenomenon was noted using hypoxia-reoxygenation injury, which induced a greater than 30% release of cellular LDH from WT hepatocytes (Fig. 8E). *Ninj1^−/−^* hepatocytes had substantial protection against this injury, and glycine was able to phenocopy this degree of protection in WT hepatocytes. To confirm that the cellular protection is due strictly to NINJ1 inhibition, the activation of caspase-1, a protease upstream of NINJ1 induced in pyroptosis, we used a fluorochrome-labeled inhibitor of caspase-1 (FLICA) assay. Hypoxia-reoxygenation induced caspase-1 cleavage in hepatocytes, which was not prevented by glycine pretreatment (fig. S9). This demonstrates that the effect of NINJ1 inhibition does not disrupt upstream elements of the pyroptosis pathway.

Our in vitro data indicate that both Kupffer cells and hepatocytes express NINJ1, which executes membrane rupture in response to hypoxia-reoxygenation. These findings are consistent with the protection observed in our cell type–specific animals (Fig. 7).

## DISCUSSION

IRI of the liver allograft during transplant is associated with patient morbidity and mortality and worse overall outcome. Lytic cell death is a primary driver of the acute injury process, with NINJ1 positioned as the essential terminal effector of plasma membrane rupture (*17*) across multiple forms of lytic cell death relevant to hepatic IRI, including pyroptosis and ferroptosis (*10*, *15*). We previously demonstrated that NINJ1 inhibition with a neutralizing antibody abrogated several in vivo models of acute liver injury, including diminishing plasma markers rather than histopathologic indicators of IRI-induced hepatocellular injury in mice (*25*). In the current study, we address key knowledge gaps that remain. We first addressed the relevance of NINJ1 to human liver biology in the context of transplantation. By scRNA-seq, spatial transcriptomics, and RNAscope, *NINJ1* expression was predominantly seen within hepatocyte and Kupffer cell populations. Examining PR samples of transplanted human livers used for transplant revealed increased NINJ1 activation in patients with evidence of severe early allograft dysfunction.

Using *Ninj1^−/−^* KO mice and rats, we established that NINJ1 deletion results in markedly reduced serum markers of hepatocellular injury and inflammation, tissue necrosis, and cell death following IRI. Pharmacologic inhibition of NINJ1 with glycine phenocopies NINJ1 deletion in vivo and in vitro. Nevertheless, *Ninj1* deletion or its pharmacologic inhibition provides significant but incomplete protection against IRI. NINJ1 mediates plasma membrane rupture in multiple cell death pathways relevant to IRI, such as pyroptosis. There are other lytic cell death pathways in which even if NINJ1 is removed, and alternative mechanisms are in place to execute cell rupture (e.g., MLKL in necroptosis) (*17*). It is also plausible that NINJ1-deficient cells that are not effectively cleared through efferocytosis ultimately succumb to a plasma membrane rupture event by passive and/or mechanical disruption. We speculate that while NINJ1 inhibition offers notable protection, it does not guard against all possible mechanisms of cellular rupture. Therefore, the release of biochemical markers still occurs but to a lesser extent.

In the murine liver, NINJ1 is similarly expressed by Kupffer cells and hepatocytes and mediates plasma membrane rupture in both cell types in response to cytotoxic signals, including hypoxia-reoxygenation. In vivo, both hepatocyte- and macrophage-specific deletion of *Ninj1* decrease IRI, suggesting that both cell types play a role. Hepatocyte-specific *Ninj1* KO limited IRI as evidenced by decreased circulating transaminase levels and lower Suzuki score. However, the extent of confluent necrosis was similar between WT and KO animals. The extent of necrotic tissue does not capture scattered hepatocyte injury and death. It is plausible that the conditional hepatocyte *Ninj1* KO protects a population of hepatocytes from rupture that is distributed more diffusely throughout the ischemic and reperfused tissue. These hepatocytes in the WT would contribute to the total AST and ALT release, whereas they may not in the hepatocyte-specific *Ninj1* KO. The areas of confluent necrosis, which represents severely injured tissue, could remain independent of *Ninj1* genotype. The macrophage-specific *Ninj1* KO was also protected from the IRI as evidenced by decreased circulating transaminases, confluent necrosis, and Suzuki score. This suggests that NINJ1-mediated rupture in the macrophage compartment can also drive the extent of hepatocyte injury. In this way, the similar confluent necrosis scores in the WT and hepatocyte-specific *Ninj1* KO animals could partly reflect the macrophage-driven NINJ1 contribution to injury. These findings are consistent with our in vitro observation that Kupffer cells are susceptible to hypoxia-reoxygenation and undergo a NINJ1-mediated lytic cell death. The released intracellular content from the macrophages likely propagates inflammation and may also release toxic molecules to surrounding hepatocytes. In this model, the Kupffer cell intracellular content could exacerbate the injury experienced by the hepatocyte during IRI. This would be evidenced by increased circulating transaminases, LDH release, and necrosis as we observed. By limiting the inflammatory insult and potential injurious intracellular molecules, *Ninj1* KO specifically in the Kupffer cells would partially mitigate the extent of hepatocyte injury. Overall, these findings reveal that NINJ1 plays a crucial role in mediating plasma membrane rupture during IRI across cell types and species, offering a potential therapeutic target to promote organ health in liver transplant.

Our in vivo model of IRI uses a 1-hour period of ischemia followed by an acute period of 6 hours of reperfusion. With these conditions, the hepatocyte compartment experiences a significant injury as evidenced by the increases in serum markers of hepatocyte injury. A recent study using bone marrow adoptive transfer to generate chimeric animals in which *Ninj1* is knocked out of the bone marrow compartment evaluated the effect of 1 hour of liver ischemia and either 6 or 24 hours or reperfusion (*49*). *Ninj1* KO in the bone marrow compartment alone did not affect IRI injury in their model after 24 hours of reperfusion (e.g., AST, ALT, tissue necrosis, and TUNEL-positive nuclei), whereas we observed markedly reduced indices of liver injury in our whole-body *Ninj1* KO and glycine-treated animals at 6 hours. Hu *et al.* depleted macrophages prior to bone marrow transplant by treating the mice with liposomal clodronate (*49*). Although this approach does deplete macrophages, including Kupffer cells, clodronate liposomes may primarily affect the neutrophil compartment (*50*). We observed a comparable neutrophil recruitment to the injured liver between *Ninj1* WT and KO animals, which likely reflects that the chemokine and cytokines responsible for neutrophil chemotaxis to the site of injury are upstream of cellular rupture and DAMP release. However, once recruited, the neutrophils may not behave with the same response in the setting of limited cellular lysis (*Ninj1* KO and decreased DAMP release) as compared with the WT condition. Furthermore, in clodronate-treated animals, hepatocytes retained NINJ1 expression, which may contribute to some of the differences in findings. Nevertheless, combined, our study and that of Hu *et al.* (*49*) suggest that NINJ1 activity is relevant in different liver cell populations at different phases of the IRI insult. Hence, it will be important to evaluate the long-term impact of NINJ1 KO or inhibition following IRI. This could be accomplished by using an orthotopic liver transplantation model in the *Ninj1* KO rats to understand whether inhibiting NINJ1 and thereby limiting the early inflammatory response during IRI will affect the chronic sequelae of liver transplantation, such as fibrosis and graft rejection. This model would similarly provide opportunity to delineate the importance of NINJ1 in the donor graft and recipient, respectively.

Mechanistically, our findings are consistent with a model in which NINJ1 inhibition prevents plasma membrane rupture in liver cells terminally injured by the ischemia-reperfusion insult. Plasma membrane rupture results in the release of intracellular contents, including DAMPs. Although smaller DAMPs (e.g., adenosine 5′-triphosphate) can efflux through small membrane pores, larger ones [e.g., high mobility group box 1 (HMGB1)] require membrane rupture to be released into the extracellular environment (*51*, *52*). DAMPs, such as HMGB1, are known to contribute to hepatic IRI including in the context of liver transplantation (*53*–*56*). In addition to proteins, mitochondrial DNA (mtDNA) has also been implicated as an important DAMP in sterile inflammatory responses (*57*). mtDNA levels correlate with donor liver injury and primary graft dysfunction (*58*–*60*). Our data indicate that targeting NINJ1 only inhibits cell rupture; upstream pathways, including caspase-1 activation in pyroptosis, remain intact. These upstream pathways produce their own distinct inflammatory mediators (e.g., interleukin-1 cytokines) whose release does not require membrane rupture. The release of these DAMPs through cellular rupture involves NINJ1 oligomerization into multimeric complexes that act to disrupt the plasma membrane (*22*–*24*). The activation trigger for NINJ1 polymerization, particularly within the injured liver, is not fully elucidated. Recent studies suggest that plasma membrane strain or cell swelling as seen during the cell death process initiates NINJ1 oligomerization (*19*, *61*). How molecular and biophysical changes in the plasma membrane are transmitted to NINJ1 requires further investigation

While we have focused our investigations on the role of NINJ1 in executing plasma membrane rupture during liver injury, NINJ1 was first described as a cell-cell adhesion molecule as mediated by a putative cell adhesion domain within its N terminus (*20*, *62*). As a cell adhesion molecule, it is plausible that NINJ1 enables recruitment of immune cells to the injured tissue. In our mouse model, however, we did not observe any differences in neutrophil and macrophage cell numbers in the *Ninj1* KO animals as compared with their WT counterparts. Nevertheless, our data do not preclude the possibility that the protective effect of NINJ1 inhibition is mediated in part by suppressing cell-cell adhesion and therefore immune cell recruitment. The extent to which NINJ1 serves as a cell adhesion molecule merits re-evaluation, given the recent structural study that positions the NINJ1 N terminus within the intracellular space (*31*).

Liver transplantation is the only curative treatment for end-stage acute and chronic liver disease, but there remains a critical shortage of suitable organs for transplant (*63*). IRI during liver transplantation is a major clinical challenge that contributes to early allograft dysfunction with increased recipient morbidity or mortality (*64*). Expanding the donor pool to include more marginal organs (steatotic livers, livers procured from older donors or from donation after cardiocirculatory death) increases the risk of severe IRI and early graft dysfunction, as these organs are at higher risk (*64*–*66*). Developing novel approaches to reduce IRI may help expand the donor pool. Our ability to link glycine’s cytoprotective effect to NINJ1 activation and protection against IRI has potential translational implications to this end. Glycine has long been known to suppress reperfusion injury when added to liver perfusates (*43*). Alternatively, inhibition of NIN1 can be achieved via a blocking antibody (clone D1), which ameliorates acute liver injury across multiple mouse models, including IRI (*25*). Clone D1 pretreatment significantly reduces circulating levels of hepatic transaminases 6 hours post–reperfusion injury, although a significant difference in tissue necrosis was not observed (*25*). This difference in efficacy between the D1 pharmacologic intervention and our knockout studies may reflect incomplete inhibition by the D1 antibody due to the dosing regimen used in our early study. Regardless, anti-NINJ1 therapies could be applied to liver perfusates during the ischemic phase of transplantation directly to the graft or via expanding utilization of normothermic machine perfusion technologies to enhance cellular integrity and thereby limit the extent of graft injury and dysfunction following transplantation.

## MATERIALS AND METHODS

Our study examined male and female human specimens as well as male and female animals. Similar findings are reported for both sexes.

### Human liver samples

All human liver samples were collected with institutional ethics approval from the Research Ethics Board (REB) at the University Health Network (UHN), Toronto, Canada (REB no. 22-5892) and The Hospital for Sick Children (REB no. 1000082047). Liver tissue was obtained from the UHN Multi-Organ Transplant (MOT) Biobank (REB no. CAPCR 15-9179). All adult patients (aged over 18 years) underwent liver transplantation at UHN from 2016 to 2022 using allografts from a deceased donor for which liver biopsy cryopreserved tissue specimens are stored in the MOT Biobank. Inclusion criteria for the present study were the availability of PR biopsy liver tissue and either AST <500 or >5000 within the first 7 days posttransplant for the control and early allograft dysfunction cohorts, respectively (*30*, *67*). Patients who received a live donor liver allograft or for which available clinical data were incomplete were excluded. Convenience sampling of the first available 18 patients meeting inclusion criteria was used. Patient demographic and clinical characteristics are shown in table S1.

### Single-cell RNA sequencing, spatial transcriptomics, and RNA in situ hybridization

scRNA-seq data from 24 healthy human livers were obtained from GSE234977 (*28*). The IRI scRNA-seq dataset was obtained from (*29*), comprising three perioperative time points from living-donor transplantation: PP, EP, and PR (GEO: GSE162694) (*29*). All analyses were performed in R (v4.4.1) using Seurat (v5.3.1). Cell types in the IRI scRNA-seq dataset were annotated by label transfer from the down-sampled NDD reference atlas using Seurat’s anchor-based annotation transfer. *NINJ1* expression was quantified from normalized counts, and bar graphs show mean ± SEM across donors, with individual donor-level means overlaid as points. Pairwise comparisons between IRI time points were performed by Wilcoxon rank-sum test using the ggsignif R package (v0.6.4). Visium spatial transcriptomics sections (*28*) were analyzed in Seurat, and spot-level colocalization of *NINJ1* and *GLUL* was quantified by Spearman’s rank correlation. RNAscope (Bio-Techne) was completed on paraffin-embedded human liver samples as per the manufacturer’s instructions. In situ hybridization was completed using the following probes: *CD68*-C1 probe no. 402681, *NINJ1*-C4 probe no. 540051-C4, *ALB*-C3 probe no. 600941-C3, and *VWF*-C2 probe no. 413401-C2. Images were acquired using Imaris acquisition software (Oxford Instruments; RRID: SCR_00737).

### Animals

All animal procedures were conducted under protocols approved by the Animal Care Committee at The Hospital for Sick Children (Animal Use Protocols Nos. 58856 and 685187 for mice and rats, respectively) and in accordance with animal care regulation and policies of the Canadian Council on Animal Care. Mice and rats were housed in same-sex cohorts in polycarbonate cages with ad libitum access to food and water. Housing rooms were temperature and humidity controlled with 14-hour:10-hour light:dark cycles.

#### Mice

*Ninj1* KO (*Ninj1^−/−^*) mice on a C57BL/6 background were previously described (*17*), with *Ninj1* WT littermate mice used as controls. In glycine treatment studies, WT C57BL/6 animals were purchased from the Jackson Laboratory (strain catalog no. 000664; Bar Harbor, MA, USA). *Ninj1*^fl/fl^ mice with a floxed exon 3 were described previously (*25*). Hepatocyte and macrophage-specific *Ninj1* KO mice were generated by breeding *Ninj1^fl/fl^* mice with Alb-cre [B6.Cg-Speer6-ps1^Tg(Alb-cre)21Mgn^/J; Jackson Laboratory, strain catalog no. 003574] and LyzM-cre [B6.129P2-Lyz2^tm1(cre)Ifo^/J, Jackson Laboratory, strain catalog no. 004781]. Genotyping by polymerase chain reaction (PCR) was conducted as per instructions provided by the Jackson Laboratory.

#### Rats

The *Ninj1* allele was generated by ENVIGO using established CRISPR methodology and electroporation of Cas9 in complex with single guide RNAs (sgRNAs) into Sprague Dawley rat zygotes (Taconic). Two sgRNAs were designed to generate a 368–base pair (bp) deletion at genomic coordinates 12301–12668. Non-Homologous End Joining (NHEJ) activity of the upstream sgRNA was detected using PCR primer set A (5′-tcttgctggagaccagtgtg and 5′-gaagaaggcgaactcattgc), yielding an expected band size of 396 bp. NHEJ activity of the downstream sgRNA was detected using PCR primer set B (5′-gcaatgagttcgccttcttc and 5′-gagccacagggatctaagga), yielding an expected band size of 338 bp. *Ninj1^−/−^* was genotyped with PCR primer set C (5′-tcttgctggagaccagtgtg and 5′-gagccacagggatctaagga), yielding ∼362 bp *Ninj1^−/−^*. WT rats were littermates.

### Segmental IRI model

Mixed-sex cohorts of 6- to 12-week-old mice or rats of either *Ninj1^−/−^* or WT littermate controls were used in a 70% segmental ischemia-reperfusion model (*68*). Under isoflurane anesthesia, a sagittal midline laparotomy was made, and an atraumatic vascular clamp was placed on the portal vein and the hepatic artery to block blood flow to the left and medial lobes of the liver. After 1 hour, the clamps were removed and the animal was returned to its home cage to allow for reperfusion. Following 6 hours of reperfusion, the animal was euthanized by cardiac puncture under general anesthesia, and blood and tissues were collected for analysis. Sham laparotomy, where the vascular pedicle was exposed but not clamped, was used as a negative control. Where indicated, animals were randomly allocated into either the glycine-treatment group or the valine-treatment group. Animals were treated with intraperitoneal injection 1 hour before and immediately after removal of the vascular clamps with glycine [0.5 ml of 100 mM in phosphate-buffered saline (PBS); catalog no. G7126, MilliporeSigma, Burlington, MA, USA] or valine (0.5 ml of 100 mM in PBS, catalog no. 94640, MilliporeSigma). If ischemia-reperfusion did not induce injury in both the left and medial lobes of the liver, these animals were excluded with the assumption the vascular clamping was not optimal. Each animal was defined as an experimental unit for the purpose of sample size determination. Our primary outcome was confluent necrosis as assayed by histopathology. Secondary outcomes included serum biochemistry for liver injury and cell death as assayed by TUNEL assay on pathology samples. Experimenters were not blinded to surgical intervention (sham versus IRI) but were blinded to genotype or pharmacologic treatment group allocation during the experiment and subsequent analyses. Our study adhered to the ARRIVE 2.0 guidelines for animal experimentation (*69*).

### Serum biochemistry

Blood was collected by cardiac puncture at the end of the reperfusion period and placed on ice. Serum was prepared by centrifugation of the clotted blood. A quantitative LDH colorimetric assay kit was used to measure LDH in the serum samples as per the manufacturer’s instructions (catalog no. MAK006, MilliporeSigma). Serum AST and ALT levels were measured at the Pathology Core laboratory at The Centre for Phenogenomics (Toronto, Canada) using a Beckman Coulter AU480 clinical chemistry analyzer by photometry testing (Beckman Coulter Life Sciences, Indianapolis, IN, USA) in combination with appropriate calibrators (Beckman Coulter Lyophilized Chemistry Calibrator Levels 1 and 2) and quality control materials (Liquid Assayed Multiqual 1 and 3, Bio-Rad, Hercules, CA, USA).

### Histology

All ischemic and reperfused liver lobes were collected, paraffin-embedded, and sectioned at a thickness of 4 μm. Serial sections were then stained with hematoxylin and eosin or TUNEL (Terminal Transferase, catalog no. 03 333 566 001 and Biotin-16-dUTP, catalog no. 11 093 070 910, Roche Diagnostics, Inc., Indianapolis, IN, USA). Tissue specimen processing and staining were conducted at the Spatio-Temporal Targeting and Amplification of Radiation Response (STTARR) Innovation Centre (Toronto, Canada). Slides were imaged using a Panoramic Flash II Slide Scanner (3DHistech Inc., Budapest, Hungary) and visualized using HALO Image Analysis Platform (RRID: SCR_018350; Indica Labs, Albuquerque, NM, USA). To evaluate the extent of necrosis within the collected liver samples, the DenseNet V2 classifier supervised machine learning algorithm (HALO Image Analysis Platform) was trained to recognize necrotic tissue in the H&E stains and applied to the entire liver sample. To evaluate the TUNEL-positive nuclei, we used the nuclei-seg pretrained classifier plug-in into the multiplex immunohistochemistry (IHC) module to distinguish TUNEL-stained (brown) from hematoxylin-stained (blue) nuclei. The software was trained on multiple samples prior to developing an effective generalized classifier. Negative annotation tool was used to exclude regions with high background staining on the edges or regions of poor tissue sample integrity (due to edge-effects or tears in the tissue).

### Immunohistochemistry of macrophage and neutrophils

Formalin-fixed paraffin-embedded tissue samples mounted on slides, stained with hematoxylin, were dewaxed and rehydrated using a standard protocol. Samples were also blocked for endogenous peroxidase activity in 3% H_2_O_2_. Antigen retrieval was performed in a citrate buffer (ab93678, Antigen Retrieval Buffer 100x, pH 6.0, Abcam) with heating at 98°C. Following washes, samples were protein-blocked with Dako’s Serum Free Protein Block (X0909, Dako) followed by incubation with primary antibodies: rabbit anti-Ly6G (1:200 dilution, 87048, Cell Signaling) or rabbit anti-F4/80 (1:100, catalog no. 70076S, Cell Signaling) 1:100 in Dako diluent (catalog no. S0809, Dako), and incubated for 1 hour at RT. After washes, samples were incubated with the biotinylated anti-rabbit immunoglobulin (Ig), secondary antibody (BA-1000, Vector Labs) for 30 min at room temperature (RT). Detection with avidin-biotin complex (ABC) system [Vector Labs VECTASTAIN Elite ABC-HRP Kit, Peroxidase (Standard) PK-6100] was done by incubation for 30 min at RT. The sample was next incubated for 10 min in DAB (3,3′-diaminobenzidine; ab64238 DAB Substrate Kit, Abcam) solution made according to product datasheet. After, sample dehydration was done through a graded alcohol series, and the samples were cleared in xylene and mounted with coverslips. To quantify the number of neutrophils and macrophages within the mouse liver tissue samples, we used the multiplex IHC module in HALO Image Analysis Platform to distinguish Ly6G-stained (neutrophils) and F4/80-positive (macrophages) cells.

### NINJ1, CD68, CD31, and F4/80 immunofluorescence in mouse tissue

Formalin-fixed, paraffin-embedded 4-μm liver slides of ischemic and reperfused or sham WT mice underwent deparaffinization and rehydration, following antigen-retrieval with 10 mM tris + 1 mM EDTA buffer. The slides were washed two times with Tris-buffered saline (TBS)-Tween (0.05% Tween 20) and then blocked in 10% donkey serum and 1% bovine serum albumin (BSA) in TBS-Tween for 1 hour. Slides were incubated overnight at 4°C in either anti-mouse NINJ1 primary antibody (Clone 80, provided by Genentech, Inc., South San Francisco, CA, USA) at 9 μg/ml, chicken recombinant monoclonal anti-CD68 primary antibody (1:500 dilution; catalog no. Ab318303, Abcam), rabbit monoclonal anti-CD31 primary antibody (1:500; catalog no. Ab222783, Abcam), or rat monoclonal anti-mouse F4/80 Alexa Fluor 647–conjugated (1:200; catalog no. 123122, BioLegend). The following day, the slides were washed three times with TBS-Tween and incubated in either donkey anti-mouse IgG H&L Alexa Fluor 647 (1:1000; A-3157; Thermo Fisher Scientific), donkey anti-chicken Cy3 (1:1000), or donkey anti-rabbit IgG H&L Alexa Fluor 488 (1:1000; catalog no. Ab150073, Abcam) in TBS-Tween supplemented with 1% BSA for 1 hour at RT. Following incubation, secondary antibodies were removed, and the nuclei were labeled with 4′,6-diamidino-2-phenylindole (DAPI) at 0.5 μg/ml for 5 min. Slides were then washed with TBS-Tween before being imaged by confocal microscopy on a Lecia Stellaris 8 platform with a 63× objective. Three images per treatment per experiment were taken and images were processed using ImageJ.

### Cells isolation and culturing

#### Primary macrophages

Primary BMDMs were harvested, as previously described (*26*), from the femurs and tibia of mixed-sex cohorts of either WT or *Ninj1* KO mice on a C57BL/6 background. In brief, the bones were cleaned, the ends cut, and centrifuged to collect bone marrow into sterile PBS. Following a wash in PBS, the cells were plated in Dulbecco’s modified Eagle’s medium (DMEM) with macrophage colony-stimulating factor (M-CSF) (10 ng/ml; catalog no. 315-02, Peprotech Inc., Cranbury, NJ, USA). After 5 days of culture, the BMDM were detached from the dishes with TBS with 5 mM EDTA, resuspended in fresh DMEM, and plated.

#### Primary hepatocytes and Kupffer cells

Primary mouse hepatocytes and Kupffer cells were isolated as described (*70*). In brief, under general anesthesia (2 g/kg urethane), the inferior vena cava was cannulated, and the liver was perfused to remove blood and chelate calcium using EDTA (30 ml of EDTA 0.5 mM in PBS at 300 ml/hour) before administering a Liberase collagenase digestion solution (20 ml of liberase, 45 μg/ml; LIBTM-RO, Liberase TM Research Grade, Roche Diagnostics, Indianopolis, IN, USA) in Hanks’ balanced salt solution (HBSS; catalog no. 311-515-CL, Wisent Bioproducts, St-Bruno, QC, Canada) at 300 ml/hour maintained at 37°C, to dissociate the extracellular matrix. In a bio-safety cabinet, the liver was dissected in a 10-cm dish, and cells were rendered in suspension using tweezers in HBSS and then filtered through a 40-μm cell strainer and collected at 50*g* for 2 min. Hepatocytes mainly resided in the pellet and Kupffer cells remain in the supernatant and were purified using density-based separation on a 36% (v/v) Percoll gradient (catalog no. 17089101, Cytiva, Marlborough, MA, USA). Hepatocytes were seeded on 1% gelatin-coated plates in DMEM-high glucose supplemented with 2 mM l-glutamine, pyruvate (catalog no. 319-0007-CL, Wisent Bioproducts) with addition of 10% FBS and 1% pen/strep for 3 hours, and then, the medium was changed overnight in William E medium supplemented (catalog no. W4125, MilliporeSigma, Burlington, ME, USA) with 10% FBS, sodium bicarbonate (1.5 g/liter), and 1% pen/strep. Kupffer cells were plated on gelatin-precoated plates with the same DMEM–high glucose medium used for hepatocytes, which was also supplemented with M-CSF (10 ng/ml). Primary cells were used within 48 hours of isolation.

### Pyroptosis induction

Hepatocytes were grown in DMEM low glucose with 0.5% FBS and primed with (0.5 μg/ml) LPS from *Escherichia coli* serotype 055:B5 (catalog no. L5418, MilliporeSigma), which was reconstituted at a stock concentration of 1 mg/ml. After 3 hours of LPS priming, pyroptosis was induced by addition of 20 μM nigericin (catalog no. N7143; stock 10 mM in ethanol, MilliporeSigma) for 120 min, as indicated. Where indicated, cells were treated with 5 mM glycine (catalog no. G7126, MilliporeSigma) or 5 mM valine (catalog no. 94640, MilliporeSigma) following the 3 hours of LPS priming and prior to treatment with nigericin. Kupffer cells were cultured under identical medium conditions and primed with LPS (0.5 μg/ml) for 3 hours. Pyroptosis was subsequently induced by treatment with 20 μM nigericin for 30 min. As described for hepatocytes, where indicated, Kupffer cells were incubated with 5 mM glycine or 5 mM valine immediately after the 3-hour LPS priming period and included with the nigericin treatment.

### Hypoxia-reoxygenation treatment

Hypoxia-reoxygenation was performed in primary cells (hepatocytes or Kuffer cells) grown in 12-well plates with DMEM low glucose with 0.1% FBS and pen/strep antibiotics based on the published protocol with modifications (*48*). Cells received a medium replacement with 1% O_2_ preincubated medium, for a 1-hour incubation in an Oxycycler (Biospherix Ltd., Parish, NY, USA) with a 1% O_2_/CO_2_ atmosphere. Afterward, the cells were switched to 21% O_2_ preincubated medium (DMEM + 0.1% FBS, pen/strep) and incubated for 5 hours in a 21% O_2_/CO_2_ normal atmosphere incubator with or without glycine (5 mM) supplementation.

### In vitro LDH release assay

Cells were seeded at 200,000 cells per well in 12-well plates. Following treatment, cell culture supernatants were collected and centrifuged for 5 min at 500*g* to eliminate cellular debris. On ice, the cell plates were washed once with cold PBS and lysed in lysis buffer provided in the LDH assay kit (catalog no. C20300, Thermo Fisher Scientific, Waltham, MA, USA) containing protease inhibitors (Pierce tablet, catalog no. A32955, Thermo Fisher Scientific). The collected supernatants and lysates were assayed for LDH using a colorimetric assay kit following the manufacturer’s instructions.

### Fluorescence microscopy

#### NINJ1 immunofluorescence

Primary hepatocytes from *Ninj1* WT and *Ninj1* KO mice were harvested as described above and cultured on glass coverslips. Cells were washed with PBS and fixed in 4% paraformaldehyde (catalog no. 15710, 16% stock, Electron Microscope Sciences, Hatfield, PA, USA) in PBS at room temperature for 15 min. The cells were washed three times with PBS and then blocked in 10% donkey serum in PBS for 1 hour. Cells were then incubated overnight at 4°C in rabbit monoclonal anti-mouse NINJ1 primary antibody (Clone 25, provided by Genentech, Inc., South San Francisco, CA, USA) at 10 μg/ml. The cells were washed three times with PBS and incubated in Cy3-conjugated donkey anti-rabbit secondary antibody (1:1000 dilution, catalog no. 711-165-152, Jackson ImmunoResearch) in PBS supplemented with 1% donkey serum for 1 hour at RT. Following incubation, secondary antibodies were removed and the nuclei were labeled with DAPI at 0.5 μg/ml for 5 min. Cells were then washed with PBS before being imaged by spinning disk confocal microscopy (Quorum Technologies Inc., Puslinch, Canada) on a Zeiss Axiovert 200M microscope with a 63× objective. Images were acquired by a CCD camera (Hamamatsu Photonics, Bridgewater, NJ, USA) driven by the Volocity software (RRID: SCR_002668, Quorum Technologies Inc. Puslinch, Canada). Three images per treatment per experiment were taken, and images were processed using ImageJ.

#### FAM-YVAD-FMK fluorescence

Caspase-1 activation was measured by confocal microscopy using the Pyroptosis FAM Caspase-1 Kit (catalog no. ICT9145, Bio-Rad Hercules, CA, USA) The cell-permeant fluorescent probe, FAM-YVAD-FMK, labels active capase-1 in live cells through the specific binding of the YVAD peptide to caspase-1 and irreversible covalent bond formation by the fluoromethyl ketone (FMK) reactive group. Excess unreacted reagent is washed out, and the fluorescent signal of the fluorescein (FAM) remaining in the cells is proportional to the caspase-1 activation. Hepatocytes were seeded on 18-mm glass coverslips in 12-well plates at 1 × 10^5^ cells per well and treated with hypoxia-reoxygenation as described above. FAM-YVAD-FMK was added to the cells for 60 min at the working dilution, and then, the cells were incubated with fixative provided with the kit, following the manufacturer’s instructions. After staining, coverslips were mounted onto glass slides using ProLong diamond antifade mounting medium (catalog no. P36965, Thermo Fisher Scientific). Cell images were collected, processed, and quantified as described above.

### SDS-PAGE and Western blotting

Cells grown in six-well plates, treated as indicated, were washed rapidly with PBS and lysed with 200 μl of RIPA containing protease inhibitors [50 mM tris HCl, 150 mM NaCl, 1.0% (v/v) NP-40, 0.25% (w/v) sodium deoxycholate, 1.0 mM EDTA, 0.1% (w/v) SDS, and 0.01% (w/v) sodium azide] at a pH of 7.4 supplemented with Pierce protease inhibitor tablet (catalog no. A32955, Thermo Fisher Scientific). Lysates were centrifugated at 14,000*g* for 30 min, and the supernatants were saved. The protein concentrations of cell lysates were measured using the Pierce BCA Protein Assay Kit (catalog no. 23227, Thermo Fisher Scientific). The lysates were adjusted to equal protein concentrations and were mixed 1:1 with 2× Laemmli sample buffer and incubated for 5 min at 95°C. Samples (10 μg of protein) were resolved using SDS-PAGE, 12 or 15% polyacrylamide gels, transferred to Immun-Blot PVDF Membrane (catalog no. 1620177, Bio-Rad), blocked with 1% BSA in TBS-Tween [150 mM NaCl, 0.1% (w/v) Tween 20 detergent, 20 mM Tris-HCl, pH 7.5], and then incubated with primary antibodies indicated according to the manufacturer’s or published recommendations. Antibodies used were anti-caspase-1 (p20) (1:1000, catalog no. AG-20B-0042-C100, AdipoGen Life Sciences, Inc. San Diego, CA, USA), anti-NINJ1 rabbit monoclonal (clone 25, 1 μg/ml) antibody (*17*); and anti-β-actin mouse monoclonal (1:1000, catalog no. A1978, MilliporeSigma). Following incubation with the primary antibodies, polyvinylidene difluoride (PVDF) membranes were washed with TBS-Tween, incubated with goat-anti-rabbit or donkey-anti-mouse horseradish peroxidase–linked secondary antibody (Jackson ImmunoResearch), and washed again before visualizing via reaction with the Novex ECL Chemiluminescent Substrate Reagent Kit (catalog no. WP20005, Thermo Fisher Scientific) and using ChemiDoc XRS+ Imaging System (Bio-Rad).

### Blue native–PAGE and Western blotting

#### Cells

BMDMs were seeded in 12-well plates. The following day, the cells were treated as indicated in the figure legends and then placed on ice. The cells were washed with PBS and then lysed in ice-cold digitonin lysis buffer (150 mM NaCl, 150 mM tris-HCl, pH 7.5, and 1% digitonin supplemented with Pierce, protease inhibitor tablet), scraped into microtubes, and incubated 10 min on ice with periodic mixing. Lysates were centrifuged at 20,800*g* for 30 min, and the supernatant was collected and protein concentration determined using the DC protein assay kit (Bio-Rad, catalog no. 5000111). Equal amounts of protein per condition were mixed with native-PAGE sample buffer prior to being resolved using NativePAGE 3–12% Bis-Tris gels (catalog no. BN1001BOX, Thermo Fisher Scientific) according to the manufacturer’s instructions.

#### Tissue

Liver tissue lysates were prepared as previously described in (*71*) with some modifications. Briefly, 20 mg of liver tissue were homogenized in 500 μl of sucrose buffer (250 mM sucrose, 20 mM imidazole/HCl, pH 7.0, supplemented with Pierce, protease inhibitor tablet) using a Teflon Potter-Elvehjem homogenizer for 20 strokes and then centrifuged for 720*g* for 5 min to remove unhomogenized material and nuclei. Supernatants were then centrifuged at 20,800g for 30 min to collect a crude plasma membrane fraction. The pellet was resuspended in 60 μl of solubilization buffer (50 mM NaCl, 50 mM imidazole, 2 mM 6-aminohexanoic acid, and 1 mM EDTA, pH 7.0), 25 μl was retained for SDS-PAGE (see below) and to the remaining 35 μl, 20 μl of 20% digitonin was added, and the samples were incubated 10 min on ice with periodic mixing. Samples were then centrifuged for 30 min at 20,800g and supernatants were retained, and a small 2-μl aliquot was removed (and diluted) for protein determination using the DC Protein Assay Kit (catalog no. 5000111, Bio-Rad). Next, 5 μl of 50% glycerol and 10 μl of 5% (w/v) Coomassie G-250 were added to the samples. Protein samples (10 μg) were resolved on NativePAGE 3–12% Bis-Tris gels using an XCell SureLock Mini-Cell electrophoresis system (EI0001, Thermo Fisher Scientific) according to the manufacturer’s instructions. In one of the gel lanes, the NativeMark Unstained Protein Standard (LC0725, Thermo Fisher Scientific) was loaded. After performing the electrophoresis, the gels were electrotransferred to PVDF membranes and were Western blotted (as described above) with anti-NINJ1 rabbit monoclonal (clone 25) antibody at 10 μg/ml. For SDS-PAGE experiments to assess total NINJ1 in the homogenized liver tissue from above, an aliquot was used to determine protein concentration [with the Pierce BCA Protein Assay Kit (catalog no. 23227, Thermo Fisher Scientific)], and 10 μg of samples was mixed 1:1 with 2× Laemmli sample buffer and separated on a 12% SDS-PAGE gel and Western blotted with anti-NINJ1 rabbit monoclonal antibody (clone 25, 1 μg/ml) and anti-β-actin (1:1000) as described in the Western blotting protocol.

### Quantification and statistics

Statistical testing was calculated using Prism 9.0 (GraphPad Software Inc., La Jolla, CA; RRID:SCR_002798). Data are provided as means ± SEM. Groups were compared using Student’s *t* test for two groups and analysis of variance (ANOVA) for three or more groups with Šidák’s test for multiple comparisons. All collected data were analyzed, and a *P* value of <0.05 was considered statistically significant. For nonquantitative data (e.g., Western blots), experiments were replicated three times with representative images provided in the figures.

## Supplementary Materials

### This PDF file includes:

Figs. S1 to S9 Table S1 References
