# Supplementary material for: Ninjurin-1 mediates hepatic ischemia-reperfusion injury
Source: Sci Adv. 2026 Aug 26;12(35):eaeg9496. doi: 10.1126/sciadv.aeg9496 (PMC13510749; doi:10.1126/sciadv.aeg9496)
Supplement: Supplementary file 1 [file sciadv.aeg9496_sm.pdf]

Science Advances
AAAS

Supplementary Materials for

Ninjurin-1 mediates hepatic ischemia-reperfusion injury

Jan Mossemann *et al.*

Corresponding author: Blayne A. Sayed, blayne.sayed@sickkids.ca

Sci. Adv. **12**, eaeg9496 (2026)
DOI: 10.1126/sciadv.aeg9496

This PDF file includes:

Figs. S1 to S9
Table S1
References

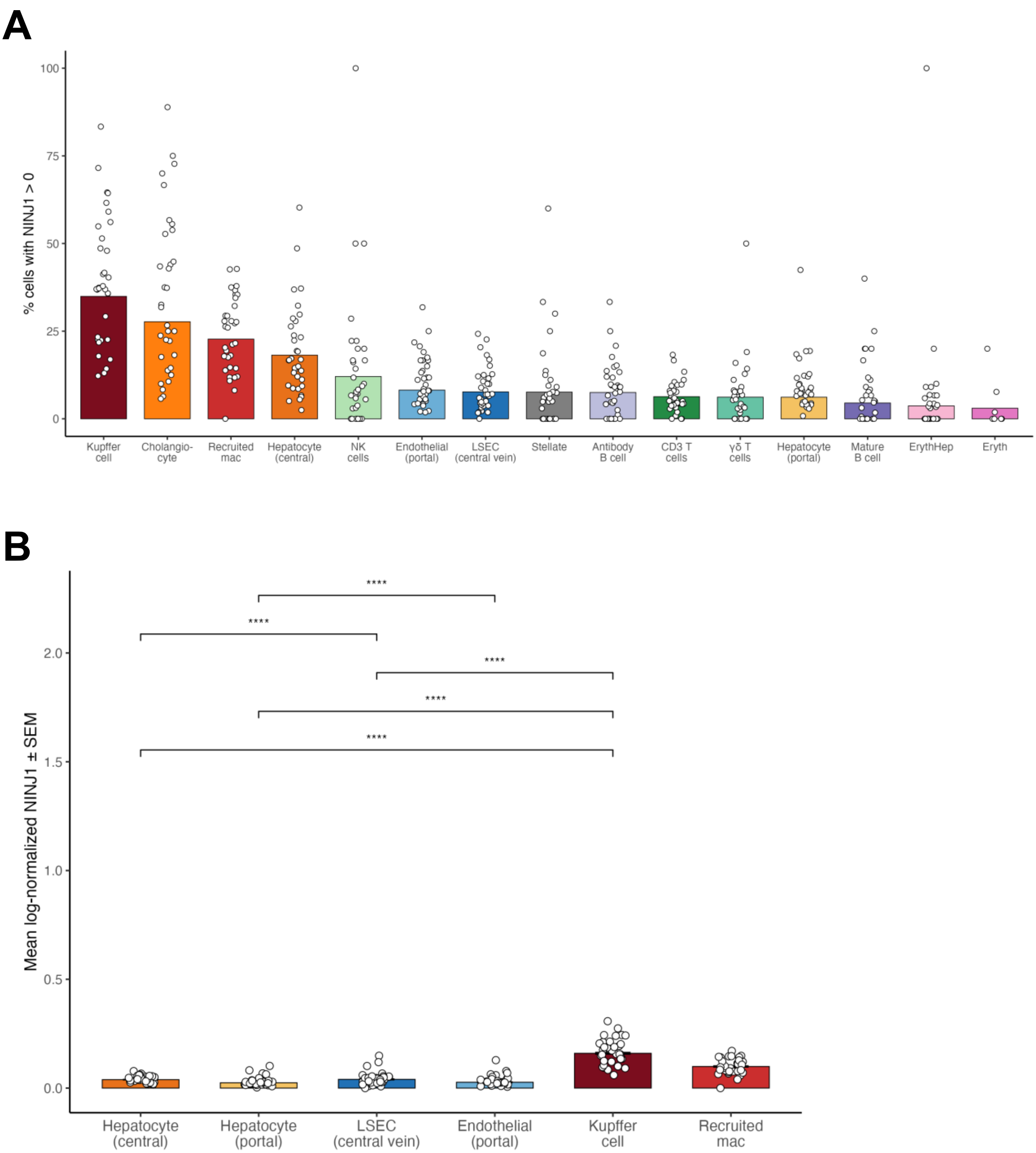

Supplemental Figure 1. NINJ1 expression is broadly expressed in the liver with a relative enrichment in Kupffer cell/macrophage populations. (**A**) Percentage of cells expressing NINJ1 (count > 0) per annotated cell type across 24 NDD liver samples (*28*). Bars represent mean with individual donors displayed as circles. (**B**) Mean log-normalized *NINJ1* expression ± SEM in central hepatocytes (CentralHep), portal hepatocytes (PortalHep), central vein liver sinusoidal endothelial cells (cvLSECs), portal endothelial cells (PortalEndo), Kupffer cells, and recruited macropahges. Statistical analysis performed by Wilcoxon rank-sum test with Bonferroni correction. *p < 0.05, **p < 0.01, ***p < 0.001, ****p < 0.0001.

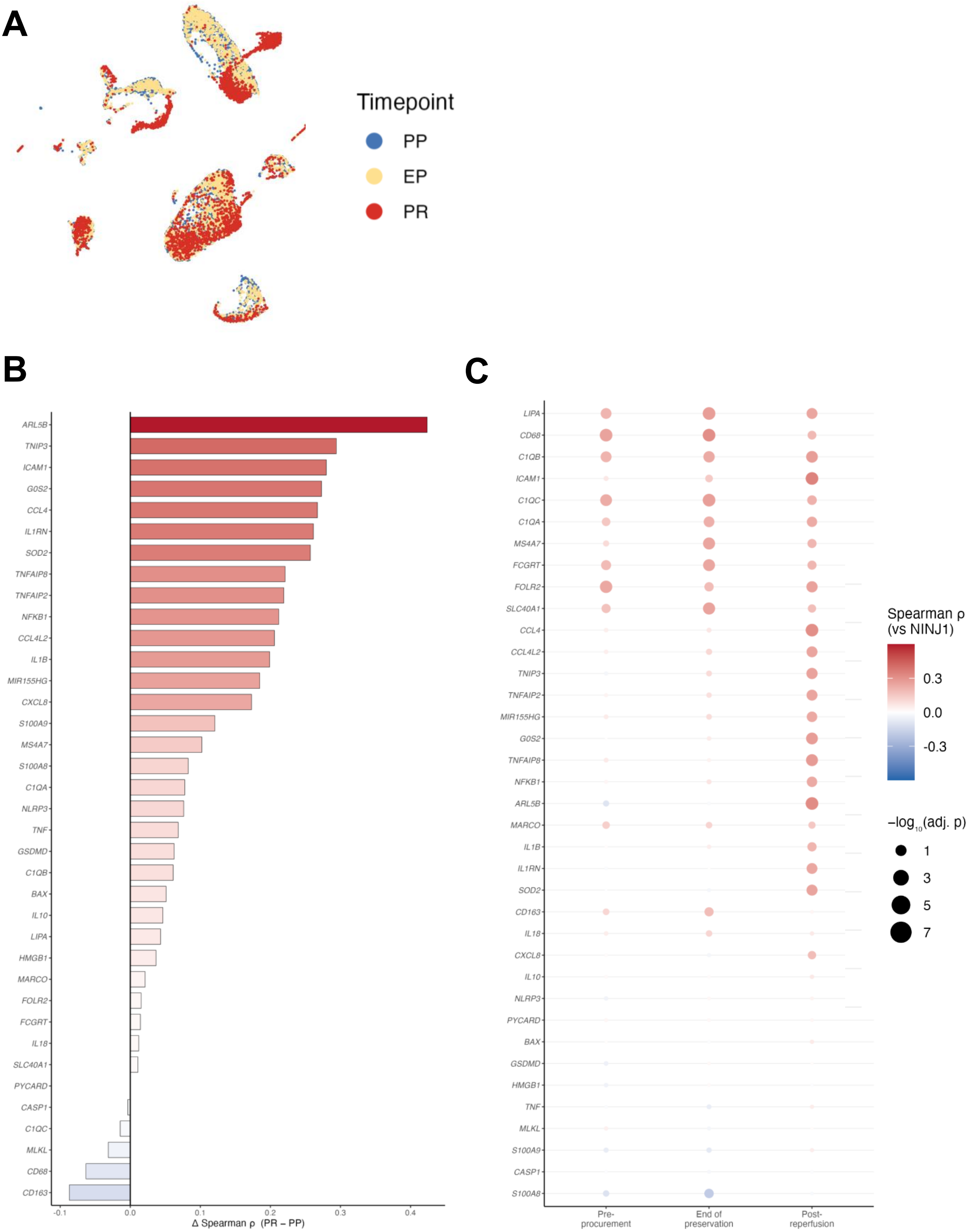
A
Timepoint
PP
EP
PR
B
ARL5B
TNIP3
ICAM1
G0S2
CCL4
IL1RN
SOD2
TNFAIP8
TNFAIP2
NFKB1
CCL4L2
IL1B
MIR155HG
CXCL8
S100A9
MS4A7
S100A8
C1QA
NLRP3
TNF
GSDMD
C1QB
BAX
IL10
LIPA
HMGB1
MARCO
FOLR2
FCGRT
IL18
SLC40A1
PYCARD
CASP1
C1QC
MLKL
CD68
CD163
-0.1
0.0
0.1
0.2
0.3
0.4
Δ Spearman ρ (PR – PP)
Positive values: genes that become more co-expressed with NINJ1 post-reperfusion
C
LIPA
CD68
C1QB
ICAM1
C1QC
C1QA
MS4A7
FCGRT
FOLR2
SLC40A1
CCL4
CCL4L2
TNIP3
TNFAIP2
MIR155HG
G0S2
TNFAIP8
NFKB1
ARL5B
MARCO
IL1B
IL1RN
SOD2
CD163
IL18
CXCL8
IL10
NLRP3
PYCARD
BAX
GSDMD
HMGB1
TNF
MLKL
S100A9
CASP1
S100A8
Pre-procurement
End of preservation
Post-reperfusion
Spearman ρ (vs NINJ1)
0.3
0.0
-0.3
−log10(adj. p)
1
3
5
7

Supplemental Figure 2. NINJ1 expression is associated with the upregulation of pro-inflammatory and injury-associated genes in Kupffer cells during human hepatic ischemia-reperfusion injury. (**A**) UMAP of 14,311 cells from (*29*) colored by timepoint: pre-procurement (PP), end of preservation (EP), 2 h post-reperfusion (PR). (**B**) Change in *NINJ1* co-expression in Kupffer cells between post-reperfusion and pre-procurement (*29*). Positive values indicate genes that become more co-expressed with *NINJ1* post-reperfusion. (**C**) Spearman correlation of *NINJ1* expression with co-expressed genes in Kupffer cells across IRI timepoints (*29*).

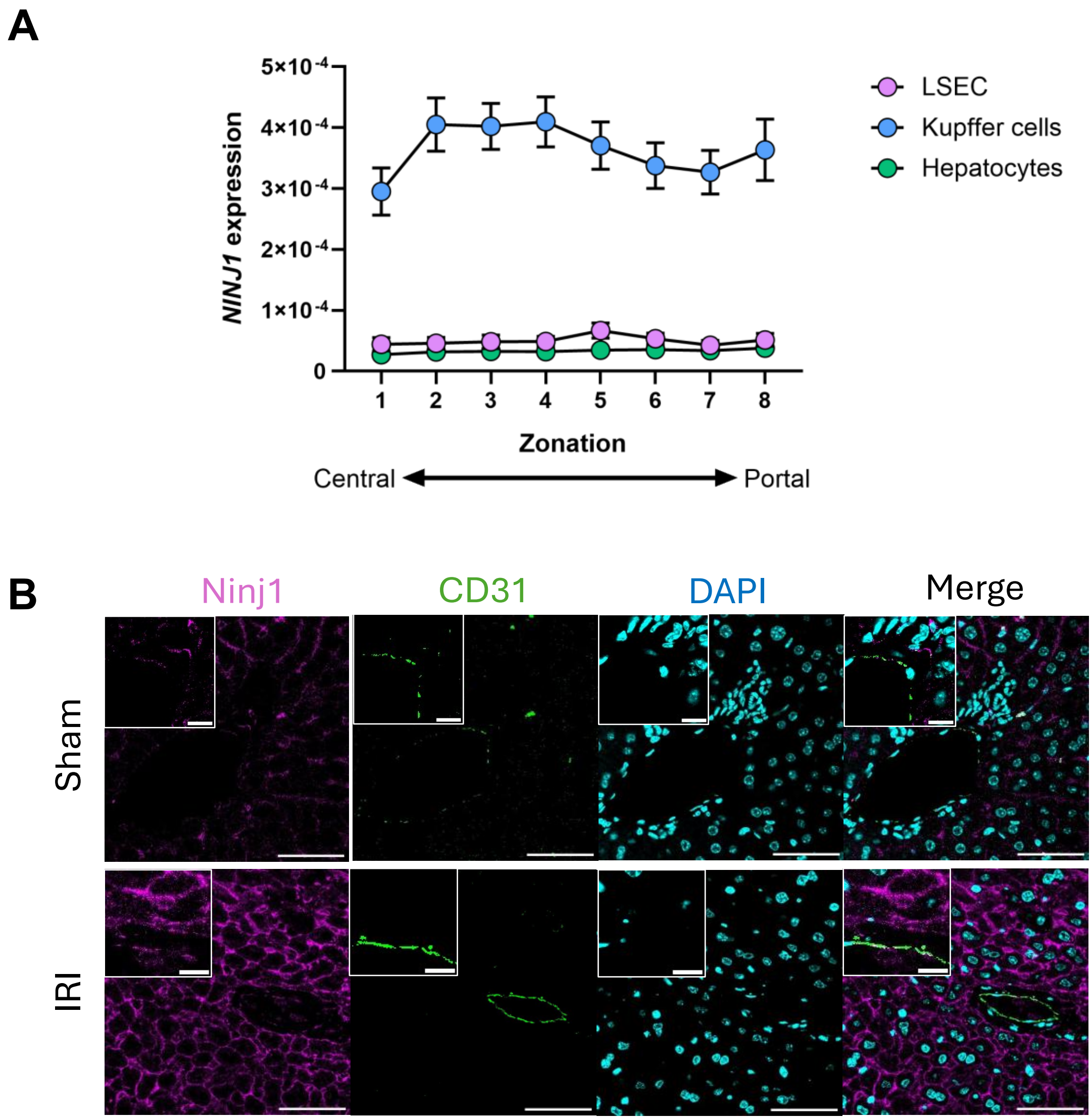

Supplemental Figure 3. NINJ1 is expressed in human and mouse liver endothelial cells. (**A**) Validation of human liver endothelial cell *NINJ1* expression using a distinct spatial transcriptomic atlas of healthy human liver (*72*). *NINJ1* is expressed in the LSEC population to a similar level of the hepatocytes, approximately 10-fold less than in Kupffer cells, and without a specific zonation pattern. (**B**) Mixed-sex cohorts of wild-type mice underwent ischemia-reperfusion injury (IRI) consisting of 1 hour of 70% segmental warm ischemia followed by 6 hours of reperfusion prior to tissue collection. Sham laparotomy was used as a negative control. Representative immunofluorescence of NINJ1 (magenta) in liver tissue from sham-operated or IRI liver. Liver endothelial cells were identified by CD31 (green). Scale bars are 50 microns and 10 microns for the full fields of view and insets, respectively.

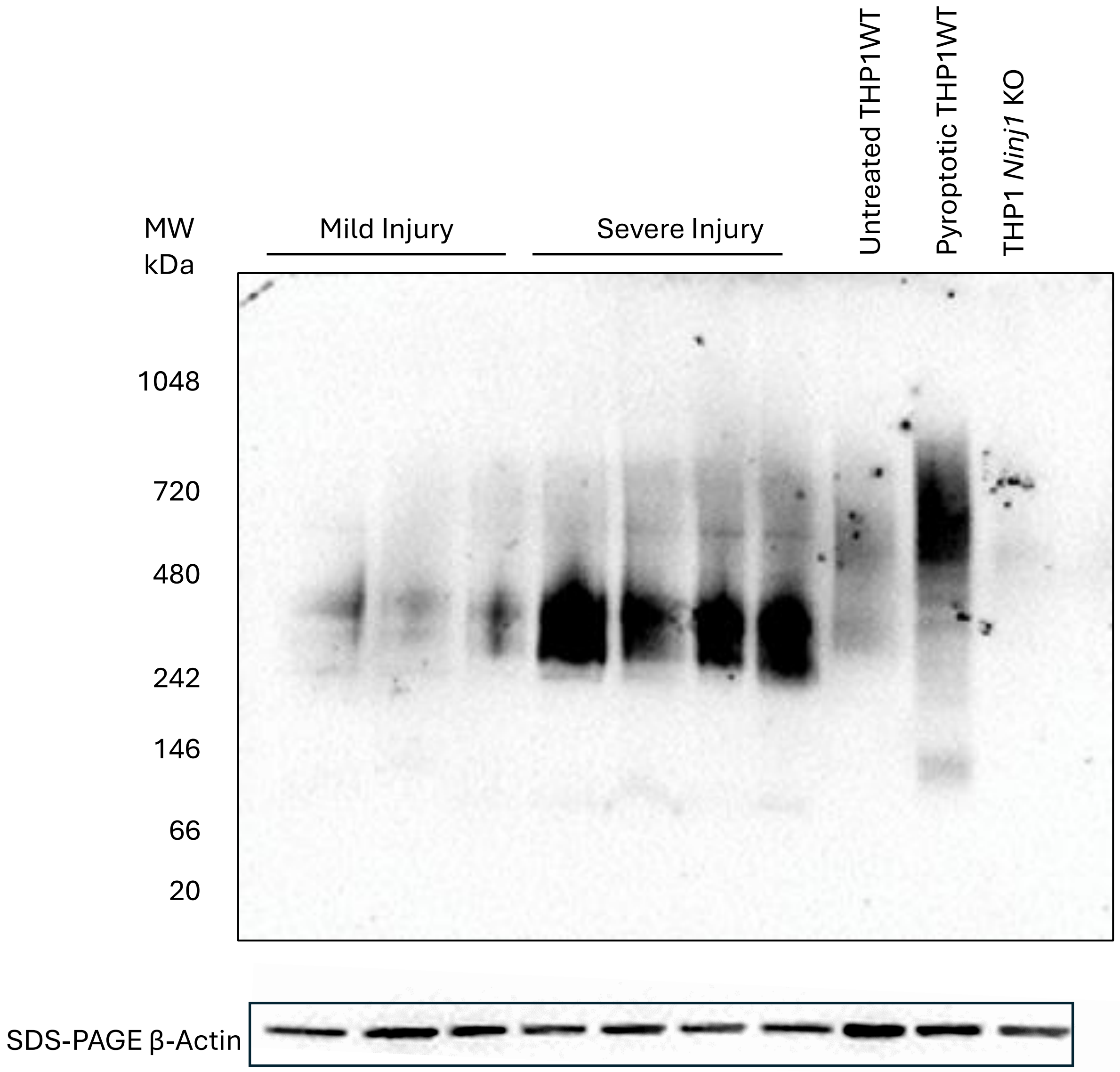

Supplemental Figure 4. Representative blue native-PAGE of human NINJ1 in patient liver samples from low and high early allograft dysfunction groups. Human liver samples from patients with mild injury (lanes 1–3) and severe injury (lanes 4–7; defined by AST > 5000 U/L) were subjected to BN-PAGE to assess NINJ1 clustering. Increased high–molecular weight NINJ1 complexes (≈242–480 kDa) were predominantly observed in the severe injury group. THP1 wild-type cell lysates (untreated), THP1 wild-type cell lysates under pyroptosis (priming with 0.5 μg/ml LPS from E. coli serotype 055:B5 for 3 hours followed by 20 μM nigericin treatment for 2 hours), and THP1 *NINJ1* knockout cell lysates were included as controls. Corresponding SDS-PAGE immunoblots for β-actin confirm similar protein loading.

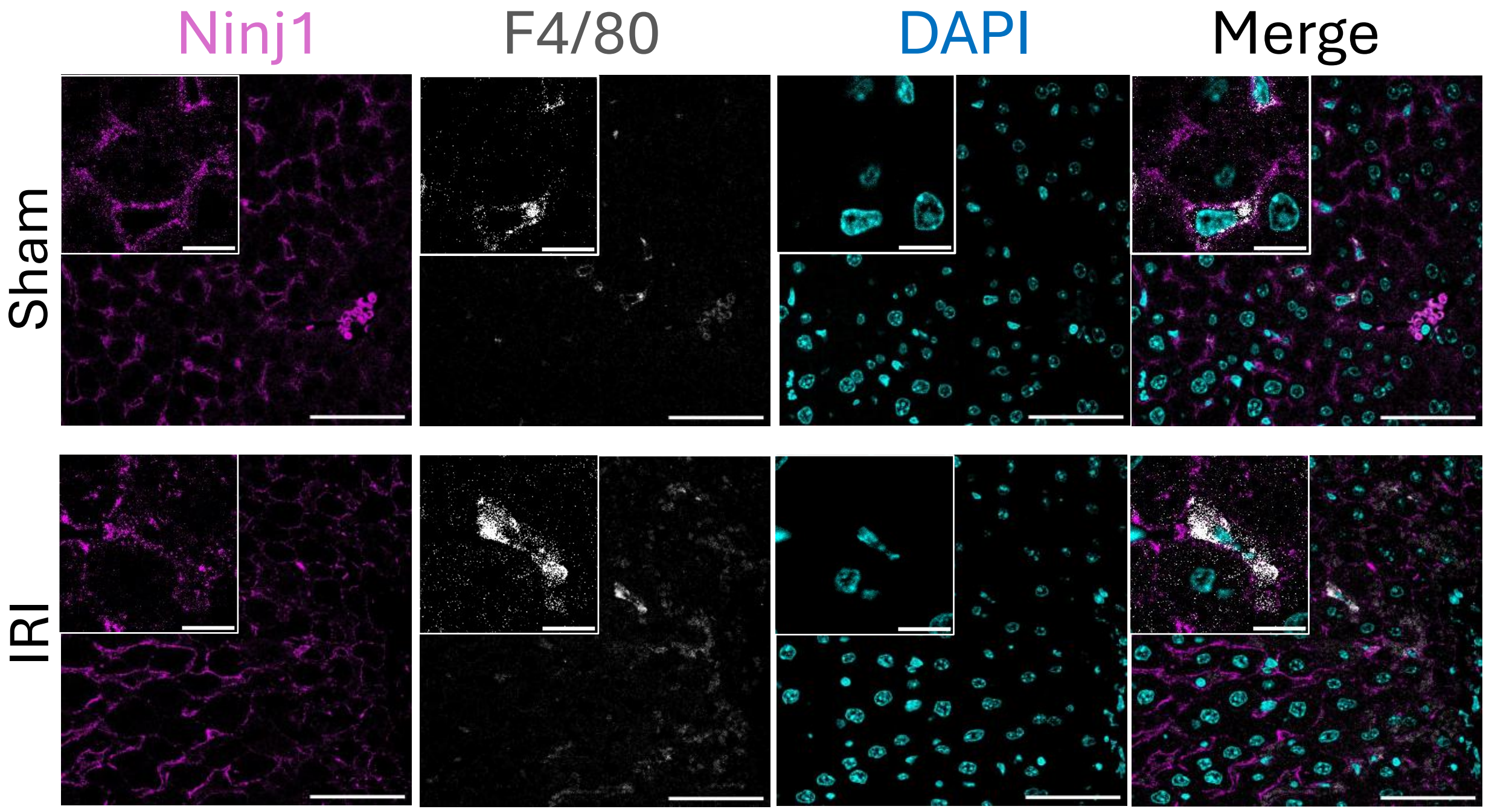

Supplemental Figure 5. NINJ1 is expressed by liver macrophages in basal and ischemia-reperfusion conditions. Mixed-sex cohorts of wild-type mice underwent ischemia-reperfusion injury (IRI) consisting of 1 hour of 70% segmental warm ischemia followed by 6 hours of reperfusion prior to tissue collection. Sham laparotomy was used as a negative control. Representative immunofluorescence of NINJ1 (magenta) in liver tissue from sham-operated or IRI liver. Liver macrophages were identified by F4/80 (grey). Scale bars are 50 microns and 10 microns for the full fields of view and insets, respectively.

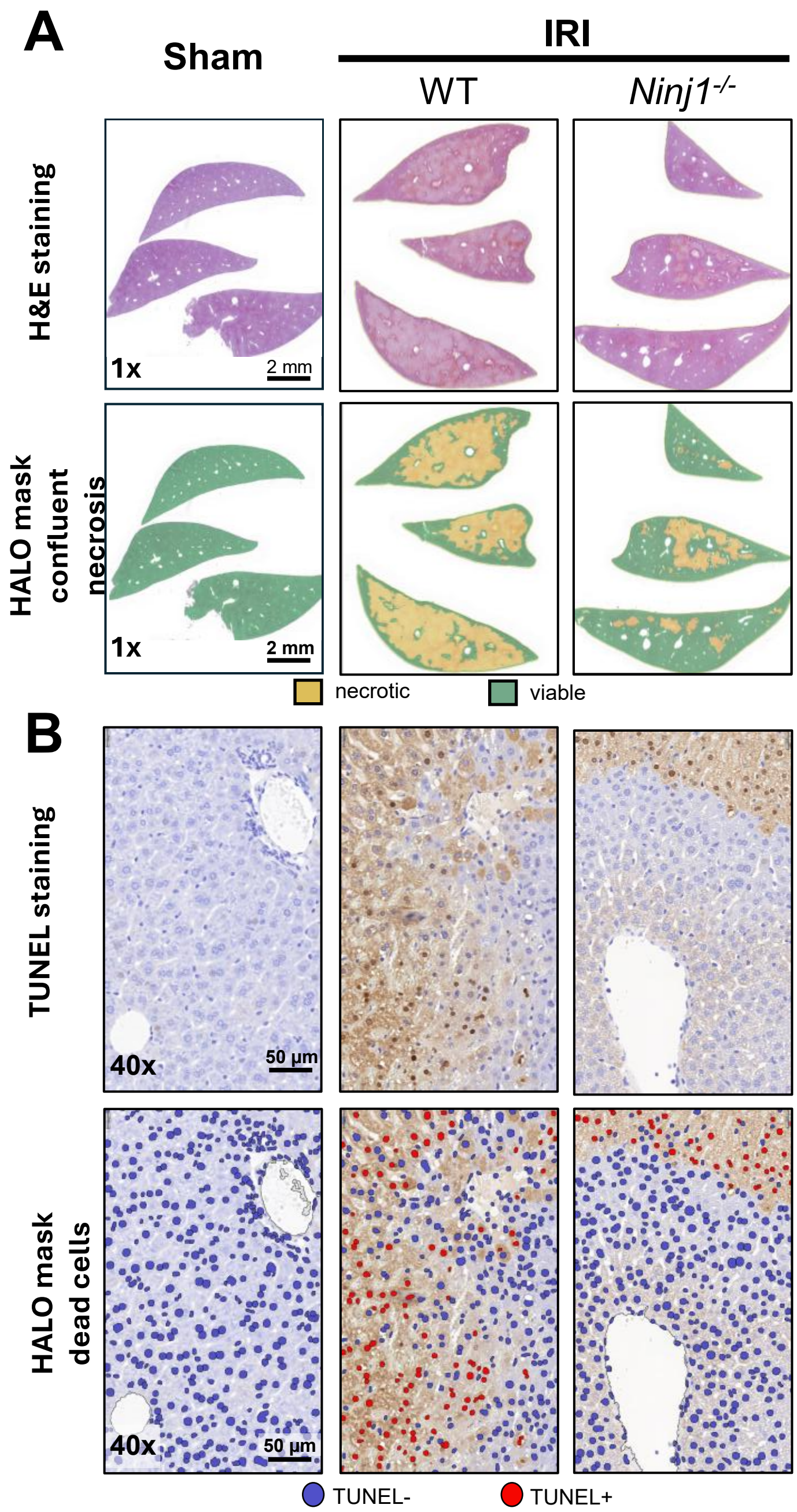
A
Sham
IRI
WT
Ninj1-/-
H&E staining
1x
2 mm
HALO mask confluent necrosis
1x
2 mm
necrotic
viable
B
TUNEL staining
40x
50 µm
HALO mask dead cells
40x
50 µm
TUNEL-
TUNEL+

Supplemental Figure 6. Examples of automated classification of necrotic liver tissue and TUNEL positive cells. As described for Figure 3, mixed-sex cohorts of *Ninj1* knockout mice or their littermate controls underwent ischemia-reperfusion injury (IRI) consisting of 1 hour of 70% segmental warm ischemia followed by 6 hours of reperfusion prior to tissue collection. Sham laparotomy was used as a negative control. Group sizes: Sham WT, N = 6; Sham KO, N = 4, IRI WT, N = 15; and IRI KO, N = 8. (**A**) To evaluate the extent of necrosis within the collected liver samples, the DenseNet V2 classifier supervised machine learning algorithm (HALO Image Analysis Platform) was trained to recognize necrotic tissue in the H&E stains and applied to the entire liver sample. Representative images of the H&E (*top*) and classified histology (*bottom*) are shown. (**B**) To evaluate the TUNEL-positive nuclei, we used the nuclei-seg pretrained classifier plug-in into the Multiplex IHC module to distinguish TUNEL-stained (brown) from Hematoxylin-stained (blue) nuclei. The software was trained on multiple samples prior to developing an effective generalized classifier. Representative images of the TUNEL stain (*top*) and classified nuclei (*bottom*) are shown and reproduced from **Fig. 4H-I** of the main text for demonstrative purposes. TUNEL negative and positive nuclei are shown in blue and red, respectively. Scale bars are as shown.

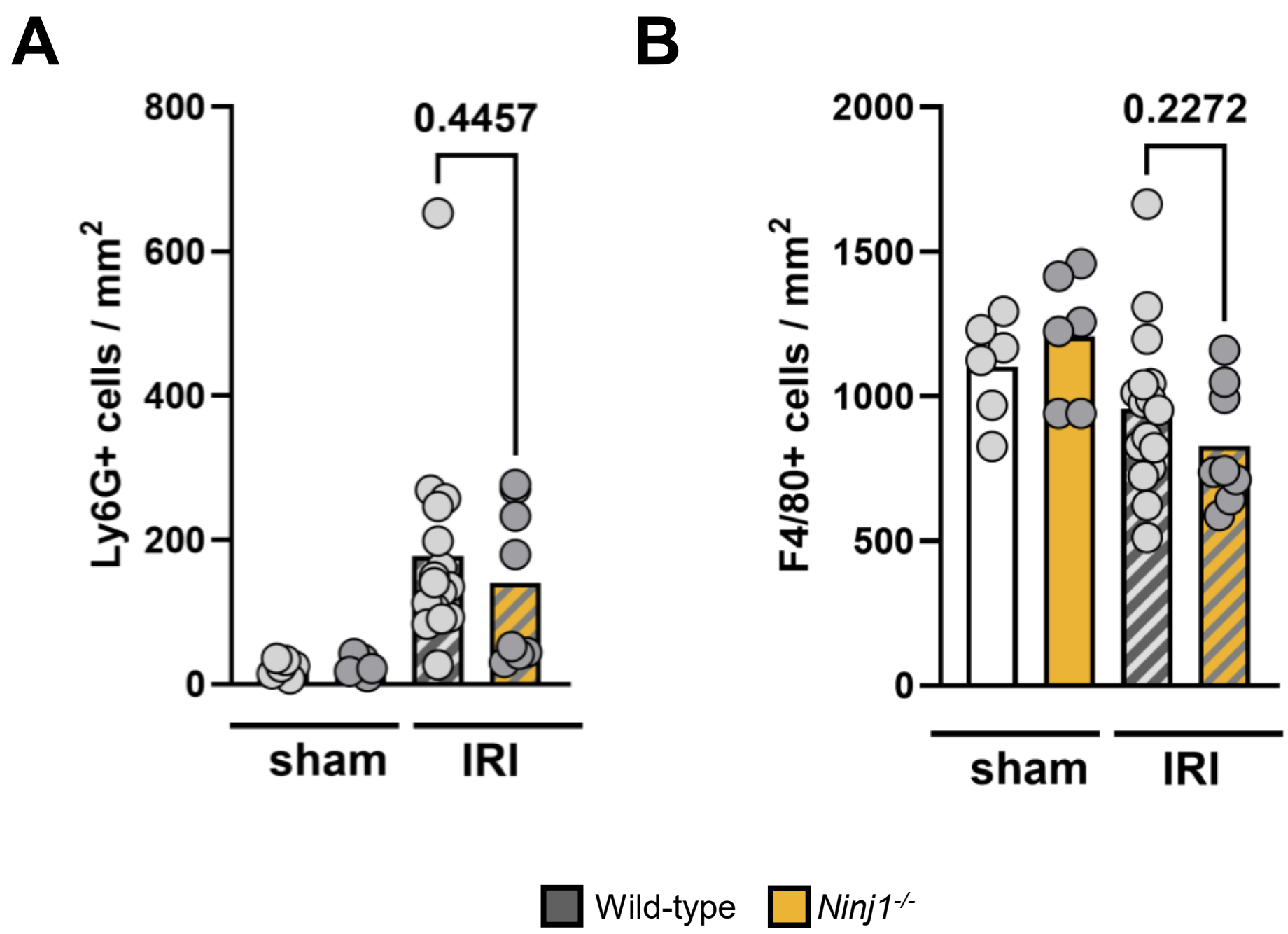

Supplemental Figure 7. Macrophage and neutrophil recruitment to the liver following ischemia-reperfusion injury in mice. Mixed-sex cohorts of *Ninj1* knockout mice or their littermate controls underwent ischemia-reperfusion injury (IRI) consisting of 1 hour of 70% segmental warm ischemia followed by 6 hours of reperfusion prior to tissue collection. Sham laparotomy was used as a negative control. Group sizes: Sham WT, N = 6; Sham KO, N = 6, IRI WT, N = 16; and IRI KO, N = 8. Liver neutrophils and macrophages were respectively quantified by counting the number of (**A**) Ly6G-positive and (**B**) F4/80-positive cells per square millimeter of liver tissue. Data points represent individual animals. *P*-values were determined by ANOVA with Šidák's multiple comparison.

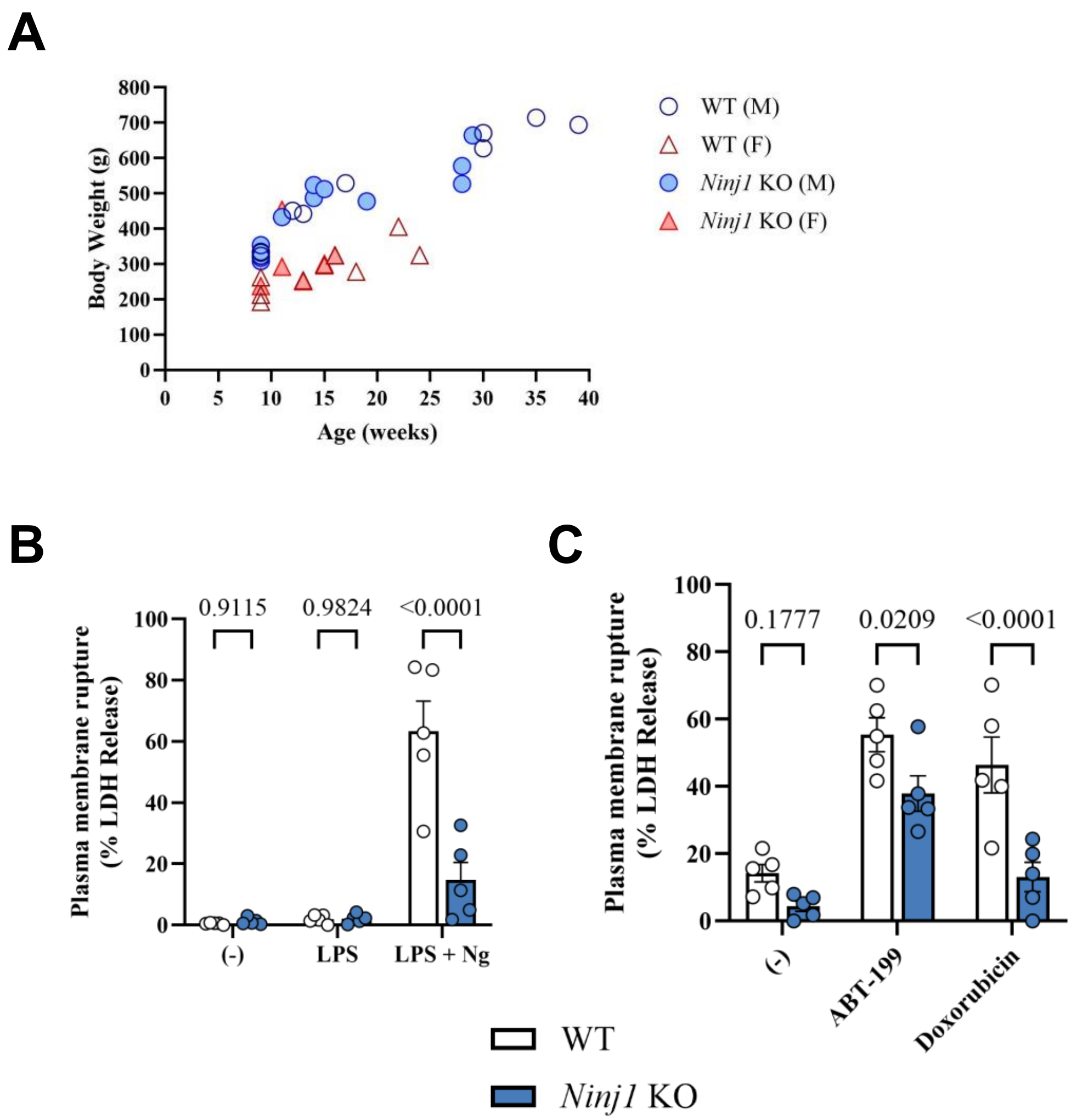

Supplemental Figure 8. Baseline characteristics of *Ninj1* knockout rats as compared to wild-type Sprague Dawley rats. (**A**) Untreated wild-type and *Ninj1* knockout male and Female rats weighed over time. Each datapoint represents an individual rat. (**B**) Bone marrow-derived macrophages were harvested from wild-type and *Ninj1* knockout rats. Pyroptosis was induced using lipopolysaccharide (100 ng/mL for 3 hours) and nigericin (20 µM for 30 min). (**C**) Secondary necrosis was induced using either ABT-199 (25 µM for 16 hours) or doxorubicin (10 µM for 16 hours). LDH release into the supernatant was measured to evaluate the extent of plasma membrane rupture. Data points represent BMDM harvested from different animals. *P*-values were determined by ANOVA.

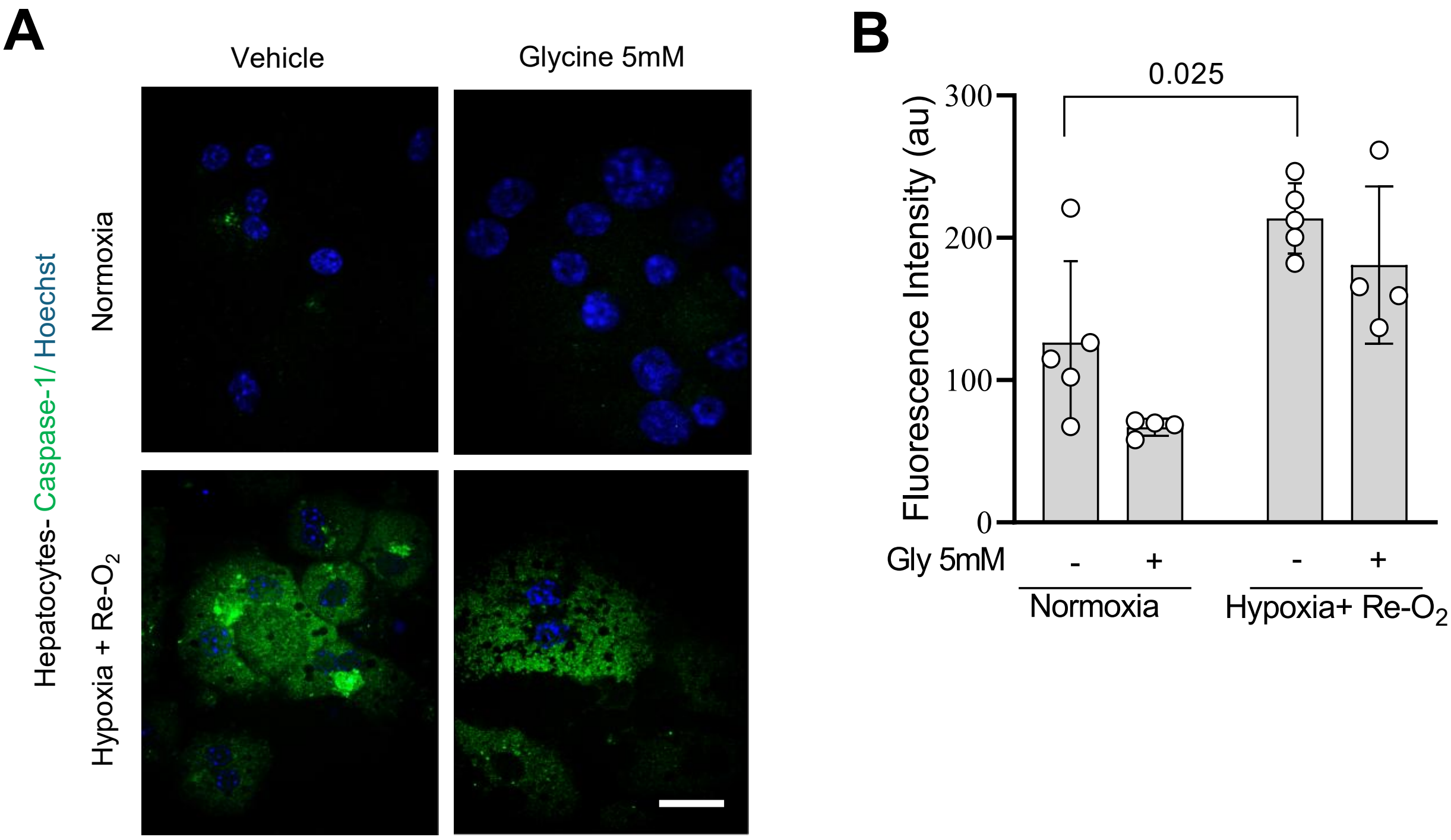

Supplemental Figure 9. NINJ1 inhibition does not affect upstream caspase-1 activation in primary mouse hepatocytes subjected to hypoxia-reoxygenation. (**A**) Representative confocal images of primary hepatocytes labeled with FLICA (FAM-YVAD-FMK) to detect active caspase-1. Under normoxic conditions (21% O_2), vehicle-treated cells and cells co-treated with glycine (5 mM) showed minimal FLICA signal. Following hypoxia–reoxygenation, hepatocytes exhibited marked caspase-1 activation, which remained detectable in both vehicle and glycine-treated groups, indicating that NINJ1 inhibition does not alter upstream caspase-1 activation. (**B**) Quantification of FLICA fluorescence intensity confirms increased caspase-1 activation under hypoxia–reoxygenation in both vehicle- and glycine-treated hepatocytes, with no reduction in response to NINJ1 inhibition.

Supplemental Table 1. Liver transplant patient demographic and clinical data.

Patient	EAD (Y/N)	Sex	MELD	Primary diagnosis	Secondary diagnosis	Donor age	Transplant warm ischemic time	Transplant cold ischemic time	AST
1	Y	Male	14	Alcoholic Cirrhosis	--	[unavailable]	72	788	32167
2	Y	Male	15	Alcoholic Cirrhosis	HPS	46	43	296	9408
3	Y	Male	26	Alcoholic Cirrhosis	--	36	42	272	7891
4	Y	Male	19	Retransplant	HCC	58	12	672	6951
5	Y	Male	7	Hepatitis C	HCC	[unavailable]	14	599	6603
6	Y	Male	8	Alcoholic Cirrhosis	HCC	53	16	335	6395
7	Y	Female	7	Hepatitis C	HCC	46	14	211	6323
8	Y	Male	29	Alcoholic Cirrhosis	--	45	57	443	7864
9	Y	Male	17	Hepatitis C	HCC	48	43	326	8654
10	Y	Male	16	Alcoholic Cirrhosis	--	23	43	234	25889
11	Y	Male	9	Alcoholic Cirrhosis	HCC	16	78	398	5197
12	N	Female	[unavailable]	α1 - Antitrypsin Deficiency	--	20	29	312	102
13	N	Male	6	Hepatitis B	HCC	21	27	301	481
14	N	Male	[unavailable]	Cirrhosis	MASH	65	13	312	162
15	N	Male	14	Hepatitis C	Alcoholic Cirrhosis	41	52	530	170
16	N	Male	34	Alcoholic Cirrhosis		69	45	273	114
17	N	Male	32	Cirrhosis	MASH	31	45	315	286
18	N	Female	13	Retransplant	PSC	18	48	312	138

Abbreviations: Aspartate transaminase, AST; Early allograft dysfunction, EAD; Hepatocellular carcinoma, HCC; Hepatopulmonary syndrome, HPS; Metabolic dysfunction-associated steatohepatitis, MASH. Early allograft dysfunction was defined as an AST value >5000 IU/mL, consistent with standard definitions (*30*, *67*).

REFERENCES

1. L. Galluzzi, I. Vitale, S. A. Aaronson, J. M. Abrams, D. Adam, P. Agostinis, E. S. Alnemri, L. Altucci, I. Amelio, D. W. Andrews, M. Annicchiarico-Petruzzelli, A. V. Antonov, E. Arama, E. H. Baehrecke, N. A. Barlev, N. G. Bazan, F. Bernassola, M. J. M. Bertrand, K. Bianchi, M. V. Blagosklonny, K. Blomgren, C. Borner, P. Boya, C. Brenner, M. Campanella, E. Candi, D. Carmona-Gutierrez, F. Cecconi, F. K.-M. Chan, N. S. Chandel, E. H. Cheng, J. E. Chipuk, J. A. Cidlowski, A. Ciechanover, G. M. Cohen, M. Conrad, J. R. Cubillos-Ruiz, P. E. Czabotar, V. D'Angiolella, T. M. Dawson, V. L. Dawson, V. De Laurenzi, R. De Maria, K.-M. Debatin, R. J. DeBerardinis, M. Deshmukh, N. Di Daniele, F. Di Virgilio, V. M. Dixit, S. J. Dixon, C. S. Duckett, B. D. Dynlacht, W. S. El-Deiry, J. W. Elrod, G. M. Fimia, S. Fulda, A. J. García-Sáez, A. D. Garg, C. Garrido, E. Gavathiotis, P. Golstein, E. Gottlieb, D. R. Green, L. A. Greene, H. Gronemeyer, A. Gross, G. Hajnoczky, J. M. Hardwick, I. S. Harris, M. O. Hengartner, C. Hetz, H. Ichijo, M. Jäättelä, B. Joseph, P. J. Jost, P. P. Juin, W. J. Kaiser, M. Karin, T. Kaufmann, O. Kepp, A. Kimchi, R. N. Kitsis, D. J. Klionsky, R. A. Knight, S. Kumar, S. W. Lee, J. J. Lemasters, B. Levine, A. Linkermann, S. A. Lipton, R. A. Lockshin, C. López-Otín, S. W. Lowe, T. Luedde, E. Lugli, M. MacFarlane, F. Madeo, M. Malewicz, W. Malorni, G. Manic, J.-C. Marine, S. J. Martin, J.-C. Martinou, J. P. Medema, P. Mehlen, P. Meier, S. Melino, E. A. Miao, J. D. Molkentin, U. M. Moll, C. Muñoz-Pinedo, S. Nagata, G. Nuñez, A. Oberst, M. Oren, M. Overholtzer, M. Pagano, T. Panaretakis, M. Pasparakis, J. M. Penninger, D. M. Pereira, S. Pervaiz, M. E. Peter, M. Piacentini, P. Pinton, J. H. M. Prehn, H. Puthalakath, G. A. Rabinovich, M. Rehm, R. Rizzuto, C. M. P. Rodrigues, D. C. Rubinsztein, T. Rudel, K. M. Ryan, E. Sayan, L. Scorrano, F. Shao, Y. Shi, J. Silke, H.-U. Simon, A. Sistigu, B. R. Stockwell, A. Strasser, G. Szabadkai, S. W. G. Tait, D. Tang, N. Tavernarakis, A. Thorburn, Y. Tsujimoto, B. Turk, T. Vanden Berghe, P. Vandenabeele, M. G. Vander Heiden, A. Villunger, H. W. Virgin, K. H. Vousden, D. Vucic, E. F. Wagner, H. Walczak, D. Wallach, Y. Wang, J. A. Wells, W. Wood, J. Yuan, Z. Zakeri, B. Zhivotovsky, L. Zitvogel, G. Melino, G. Kroemer, Molecular mechanisms of cell death: Recommendations of the Nomenclature Committee on Cell Death 2018. *Cell Death Differ.* **25**, 486–541 (2018).

2. K. Newton, A. Strasser, N. Kayagaki, V. M. Dixit, Cell death. *Cell* **187**, 235–256 (2024).

3. H. Hirao, K. Nakamura, J. W. Kupiec-Weglinski, Liver ischaemia–reperfusion injury: A new understanding of the role of innate immunity. *Nat. Rev. Gastroenterol. Hepatol.* **19**, 239–256 (2022).

4. J. Liu, K. Man, Mechanistic insight and clinical implications of ischemia/reperfusion injury post liver transplantation. *Cell. Mol. Gastroenterol. Hepatol.* **15**, 1463–1474 (2023).

5. S. Hartleif, J. Hodson, C. Lloyd, V. L. Cousin, P. Czubkowski, L. D'Antiga, D. Debray, A. Demetris, A. Di Giorgio, H. M. Evans, B. Fischler, E. Gonzales, A. S. H. Gouw, S. G. Hübscher, E. Jacquemin, F. Lacaille, S. Malenicka, V. A. McLin, M. Markiewicz-Kijewska, G. V. Mazariegos, J. K. Rajanayagam, R. Scheenstra, S. Singer, F. Smets, E. Sokal, J. E. Squires, E. Sturm, H. Verkade, D. A. Kelly, Graft Injury Group (GIG), Long-term outcome of asymptomatic patients with graft fibrosis in protocol biopsies after pediatric liver transplantation. *Transplantation* **107**, 2394–2405 (2023).

6. J. Gautheron, G. J. Gores, C. M. P. Rodrigues, Lytic cell death in metabolic liver disease. *J. Hepatol.* **73**, 394–408 (2020).

7. T. Kondo, S. Macdonald, C. Engelmann, A. Habtesion, J. Macnaughtan, G. Mehta, R. P. Mookerjee, N. Davies, M. Pavesi, R. Moreau, P. Angeli, V. Arroyo, F. Andreola, R. Jalan, The role of RIPK1 mediated cell death in acute on chronic liver failure. *Cell Death Dis.* **13**, 5 (2021).

8. E. Khanova, R. Wu, W. Wang, R. Yan, Y. Chen, S. W. French, C. Llorente, S. Q. Pan, Q. Yang, Y. Li, R. Lazaro, C. Ansong, R. D. Smith, R. Bataller, T. Morgan, B. Schnabl, H. Tsukamoto, Pyroptosis by caspase11/4-gasdermin-D pathway in alcoholic hepatitis in mice and patients. *Hepatology* **67**, 1737–1753 (2018).

9. V. L. Kolachala, C. Lopez, M. Shen, D. Shayakhmetov, N. A. Gupta, Ischemia reperfusion injury induces pyroptosis and mediates injury in steatotic liver thorough Caspase 1 activation. *Apoptosis* **26**, 361–370 (2021).

10. J. Li, J. Zhao, M. Xu, M. Li, B. Wang, X. Qu, C. Yu, H. Hang, Q. Xia, H. Wu, X. Sun, J. Gu, X. Kong, Blocking GSDMD processing in innate immune cells but not in hepatocytes protects hepatic ischemia-reperfusion injury. *Cell Death Dis.* **11**, 244 (2020).

11. M. E. Guicciardi, H. Malhi, J. L. Mott, G. J. Gores, Apoptosis and necrosis in the liver. *Compr. Physiol.* **3**, 977–1010 (2013).

12. P. Broz, P. Pelegrín, F. Shao, The gasdermins, a protein family executing cell death and inflammation. *Nat. Rev. Immunol.* **20**, 143–157 (2020).

13. W. Zhong, X. Wang, Z. Rao, X. Pan, Y. Sun, T. Jiang, P. Wang, H. Zhou, X. Wang, Aging aggravated liver ischemia and reperfusion injury by promoting hepatocyte necroptosis in an endoplasmic reticulum stress-dependent manner. *Ann. Transl. Med.* **8**, 869 (2020).

14. J. Wu, Y. Wang, R. Jiang, R. Xue, X. Yin, M. Wu, Q. Meng, Ferroptosis in liver disease: new insights into disease mechanisms. *Cell Death Discov.* **7**, 276 (2021).

15. K. Wu, T. Xu, B. Jiang, X. Yu, Y. Wang, H. Sun, Z. Zhao, M. Du, S. Wang, L. Ma, STAT1 promotes ferroptosis and inflammation in mouse hepatic ischemia-reperfusion injury. *Commun. Biol.* **8**, 1549 (2025).

16. J. Lin, M. Xu, H. Ji, W. Huang, Y. Zhou, Y. Zhou, L. Yang, Y. Sang, Y. Qian, H. Wu, X. Kong, ZBP1-sensed hypoxic stress triggers intrinsic necroptosis in hepatocytes, aggravating hepatic ischemia-reperfusion injury: An experimental study. *Int. J. Surg.* **111**, 7761–7776 (2025).

17. N. Kayagaki, O. S. Kornfeld, B. L. Lee, I. B. Stowe, K. O'Rourke, Q. Li, W. Sandoval, D. Yan, J. Kang, M. Xu, J. Zhang, W. P. Lee, B. S. McKenzie, G. Ulas, J. Payandeh, M. Roose-Girma, Z. Modrusan, R. Reja, M. Sagolla, J. D. Webster, V. Cho, T. D. Andrews, L. X. Morris, L. A. Miosge, C. C. Goodnow, E. M. Bertram, V. M. Dixit, NINJ1 mediates plasma membrane rupture during lytic cell death. *Nature* **591**, 131–136 (2021).

18. S. Ramos, E. Hartenian, J. C. Santos, P. Walch, P. Broz, NINJ1 induces plasma membrane rupture and release of damage-associated molecular pattern molecules during ferroptosis. *EMBO J.* **43**, 1164–1186 (2024).

19. Y. Dondelinger, D. Priem, J. Huyghe, T. Delanghe, P. Vandenabeele, M. J. M. Bertrand, NINJ1 is activated by cell swelling to regulate plasma membrane permeabilization during regulated necrosis. *Cell Death Dis.* **14**, 755 (2023).

20. T. Araki, J. Milbrandt, Ninjurin, a novel adhesion molecule, is induced by nerve injury and promotes axonal growth. *Neuron* **17**, 353–361 (1996).

21. C. Jennewein, R. Sowa, A. C. Faber, M. Dildey, A. von Knethen, P. Meybohm, B. Scheller, S. Dröse, K. Zacharowski, Contribution of Ninjurin1 to Toll-like receptor 4 signaling and systemic inflammation. *Am. J. Respir. Cell Mol. Biol.* **53**, 656–663 (2015).

22. M. Degen, J. C. Santos, K. Pluhackova, G. Cebrero, S. Ramos, G. Jankevicius, E. Hartenian, U. Guillerm, S. A. Mari, B. Kohl, D. J. Müller, P. Schanda, T. Maier, C. Perez, C. Sieben, P. Broz, S. Hiller, Structural basis of NINJ1-mediated plasma membrane rupture in cell death. *Nature* **618**, 1065–1071 (2023).

23. B. Sahoo, Z. Mou, W. Liu, G. Dubyak, X. Dai, How NINJ1 mediates plasma membrane rupture and why NINJ2 cannot. *Cell* **188**, 292–302.e11 (2025).

24. L. David, J. P. Borges, L. R. Hollingsworth, A. Volchuk, I. Jansen, E. Garlick, B. E. Steinberg, H. Wu, NINJ1 mediates plasma membrane rupture by cutting and releasing membrane disks. *Cell* **187**, 2224–2235.e16 (2024).

25. N. Kayagaki, I. B. Stowe, K. Alegre, I. Deshpande, S. Wu, Z. Lin, O. S. Kornfeld, B. L. Lee, J. Zhang, J. Liu, E. Suto, W. P. Lee, K. Schneider, W. Lin, D. Seshasayee, T. Bhangale, C. Chalouni, M. C. Johnson, P. Joshi, J. Mossemann, S. Zhao, D. Ali, N. M. Goldenberg, B. A. Sayed, B. E. Steinberg, K. Newton, J. D. Webster, R. L. Kelly, V. M. Dixit, Inhibiting membrane rupture with NINJ1 antibodies limits tissue injury. *Nature* **618**, 1072–1077 (2023).

26. J. P. Borges, R. S. R. Sætra, A. Volchuk, M. Bugge, P. Devant, B. Sporsheim, B. Kilburn, C. L. Evavold, J. C. Kagan, N. M. Goldenberg, T. H. Flo, B. E. Steinberg, Glycine inhibits NINJ1 membrane clustering to suppress plasma membrane rupture in cell death. *eLife* **11**, e78609 (2022).

27. A. B. den Hartigh, W. P. Loomis, M. J. Anderson, B. Frølund, S. L. Fink, Muscimol inhibits plasma membrane rupture and ninjurin-1 oligomerization during pyroptosis. *Commun. Biol.* **6**, 1010 (2023).

28. T. S. Andrews, D. Nakib, C. T. Perciani, X. Z. Ma, L. Liu, E. Winter, D. Camat, S. W. Chung, P. Lumanto, J. Manuel, S. Mangroo, B. Hansen, B. Arpinder, C. Thoeni, B. Sayed, J. Feld, A. Gehring, A. Gulamhusein, G. M. Hirschfield, A. Ricciuto, G. D. Bader, I. D. McGilvray, S. MacParland, Single-cell, single-nucleus, and spatial transcriptomics characterization of the immunological landscape in the healthy and PSC human liver. *J. Hepatol.* **80**, 730–743 (2024).

29. L. Wang, J. Li, S. He, Y. Liu, H. Chen, S. He, M. Yin, D. Zou, S. Chen, T. Luo, X. Yu, X. Wan, S. Huang, Z. Guo, X. He, Resolving the graft ischemia-reperfusion injury during liver transplantation at the single cell resolution. *Cell Death Dis.* **12**, 589 (2021).

30. B. A. Wiemann, O. Beetz, C. A. Weigle, P. Tessmer, S. Störzer, D. Kleine-Döpke, F. W. R. Vondran, N. Richter, M. Schmelzle, F. Oldhafer, Early allograft dysfunction after liver transplantation- definition, incidence and relevance in a single-centre analysis. *Langenbecks Arch. Surg.* **410**, 76 (2025).

31. S. Pourmal, M. E. Truong, M. C. Johnson, Y. Yang, L. Zhou, K. Alegre, I. B. Stowe, S. Gupta, P. A. Chen, Y. Zhang, A. Rohou, K. Newton, N. Kayagaki, V. M. Dixit, I. Deshpande, Autoinhibition of dimeric NINJ1 prevents plasma membrane rupture. *Nature* **637**, 446–452 (2024).

32. S. Suzuki, L. H. Toledo-Pereyra, F. J. Rodriguez, D. Cejalvo, Neutrophil infiltration as an important factor in liver ischemia and reperfusion injury. Modulating effects of FK506 and cyclosporine. *Transplantation* **55**, 1265–1272 (1993).

33. G. Datta, Molecular mechanisms of liver ischemia reperfusion injury: Insights from transgenic knockout models. *World J. Gastroenterol.* **19**, 1683 (2013).

34. S. L. Fink, B. T. Cookson, Apoptosis, pyroptosis, and necrosis: Mechanistic description of dead and dying eukaryotic cells. *Infect. Immun.* **73**, 1907–1916 (2005).

35. S. Yue, H. Zhou, X. Wang, R. W. Busuttil, J. W. Kupiec-Weglinski, Y. Zhai, Prolonged ischemia triggers necrotic depletion of tissue-resident macrophages to facilitate inflammatory immune activation in liver ischemia reperfusion injury. *J. Immunol.* **198**, 3588–3595 (2017).

36. M. Martignoni, G. Groothuis, R. de Kanter, Comparison of mouse and rat cytochrome P450-mediated metabolism in liver and intestine. *Drug Metab. Dispos.* **34**, 1047–1054 (2006).

37. V. O. Pustylnyak, A. N. Lebedev, L. F. Gulyaeva, V. V. Lyakhovich, N. M. Slynko, Comparative study of CYP2B induction in the liver of rats and mice by different compounds. *Life Sci.* **80**, 324–328 (2007).

38. S. Bachmann, X. X. Peng, R. T. Currin, R. G. Thurman, J. J. Lemasters, Glycine in Carolina rinse solution reduces reperfusion injury, improves graft function, and increases graft survival after rat liver transplantation. *Transplant. Proc.* **27**, 741–742 (1995).

39. F. Duenschede, S. Westermann, N. Riegler, I. Miesner, K. Erbes, P. Ewald, A. Kircher, H. Schaefer, J. Schneider, A. Schad, P. Dutkowski, A. K. Kiemer, T. Junginger, Different protection mechanisms after pretreatment with glycine or alpha-lipoic acid in a rat model of warm hepatic ischemia. *Eur. Surg. Res.* **38**, 503–512 (2006).

40. J. M. G. V. Gassner, M. Nösser, S. Moosburner, R. Horner, P. Tang, L. Wegener, D. Wyrwal, F. Claussen, R. Arsenic, J. Pratschke, I. M. Sauer, N. Raschzok, Improvement of normothermic ex vivo machine perfusion of rat liver grafts by dialysis and kupffer cell inhibition with glycine. *Liver Transpl.* **25**, 275–287 (2019).

41. H. Sheth, T. Hafez, G. K. Glantzounis, A. M. Seifalian, B. Fuller, B. R. Davidson, Glycine maintains mitochondrial activity and bile composition following warm liver ischemia-reperfusion injury. *J. Gastroenterol. Hepatol.* **26**, 194–200 (2011).

42. K. Yamanouchi, S. Eguchi, Y. Kamohara, K. Yanaga, S. Okudaira, Y. Tajima, T. Kanematsu, Glycine reduces hepatic warm ischaemia-reperfusion injury by suppressing inflammatory reactions in rats. *Liver Int.* **27**, 1249–1254 (2007).

43. Z. Zhong, S. Jones, R. G. Thurman, Glycine minimizes reperfusion injury in a low-flow, reflow liver perfusion model in the rat. *Am. J. Physiol.* **270**, G332–338 (1996).

44. W. P. Loomis, A. B. den Hartigh, B. T. Cookson, S. L. Fink, Diverse small molecules prevent macrophage lysis during pyroptosis. *Cell Death Dis.* **10**, 326 (2019).

45. J. L. Baratta, A. Ngo, B. Lopez, N. Kasabwalla, K. J. Longmuir, R. T. Robertson, Cellular organization of normal mouse liver: a histological, quantitative immunocytochemical, and fine structural analysis. *Histochem. Cell Biol.* **131**, 713–726 (2009).

46. J. D. Ellett, C. Atkinson, Z. P. Evans, Z. Amani, E. Balish, M. G. Schmidt, N. van Rooijen, R. G. Schnellmann, K. D. Chavin, Murine Kupffer cells are protective in total hepatic ischemia/reperfusion injury with bowel congestion through IL-10. *J. Immunol.* **184**, 5849–5858 (2010).

47. L. Zhang, M. Wang, R. An, J. Dai, S. Liu, M. Chen, H. Ding, Activation of NLRP3 inflammasome via Drp1 overexpression in kupffer cells aggravates ischemia-reperfusion injury in hepatic steatosis. *J. Clin. Transl. Hepatol.* **11**, 1069–1078 (2023).

48. Z. P. Evans, A. P. Palanisamy, A. G. Sutter, J. D. Ellett, V. K. Ramshesh, H. Attaway, M. G. Schmidt, R. G. Schnellmann, K. D. Chavin, Mitochondrial uncoupling protein-2 deficiency protects steatotic mouse hepatocytes from hypoxia/reoxygenation. *Am. J. Physiol. Gastrointest. Liver Physiol.* **302**, G336–342 (2012).

49. Y. Hu, F. Zhan, Y. Wang, D. Wang, H. Lu, C. Wu, Y. Xia, L. Meng, F. Zhang, X. Wang, S. Zhou, The Ninj1/Dusp1 axis contributes to liver ischemia reperfusion injury by regulating macrophage activation and neutrophil infiltration. *Cell. Mol. Gastroenterol. Hepatol.* **15**, 1071–1084 (2023).

50. S. Culemann, K. Knab, M. Euler, A. Wegner, H. Garibagaoglu, J. Ackermann, K. Fischer, D. Kienhöfer, G. Crainiciuc, J. Hahn, A. Grüneboom, F. Nimmerjahn, S. Uderhardt, A. Hidalgo, G. Schett, M. H. Hoffmann, G. Krönke, Addendum: Stunning of neutrophils accounts for the anti-inflammatory effects of clodronate liposomes. *J. Exp. Med.* **220**, e20220525 (2023).

51. A. Volchuk, A. Ye, L. Chi, B. E. Steinberg, N. M. Goldenberg, Indirect regulation of HMGB1 release by gasdermin D. *Nat. Commun.* **11**, 4561 (2020).

52. J. Schachter, A. Guijarro, D. Angosto-Bazarra, M. Pinilla, L. Hurtado-Navarro, E. Meunier, A. B. Pérez-Oliva, P. J. Schwarzbaum, P. Pelegrin, Gasdermin D mediates a fast transient release of ATP after NLRP3 inflammasome activation before ninjurin 1-induced lytic cell death. *Cell Rep.* **44**, 115233 (2025).

53. A. Tsung, R. Sahai, H. Tanaka, A. Nakao, M. P. Fink, M. T. Lotze, H. Yang, J. Li, K. J. Tracey, D. A. Geller, T. R. Billiar, The nuclear factor HMGB1 mediates hepatic injury after murine liver ischemia-reperfusion. *J. Exp. Med.* **201**, 1135–1143 (2005).

54. K. Tanaka, Y. Uchida, K. Kadono, S. Kageyama, H. Kawamoto, M. Ito, Y. Kidoguchi, K. Saga, H. Kojima, H. Hirao, K. Nakamura, K. Taura, H. Terajima, T. Watanabe, E. Hatano, Recipient toll-like receptor 4 determines the outcome of ischemia-reperfusion injury in steatotic liver transplantation in mice. *Am. J. Transplant.* **25**, 1168–1179 (2025).

55. R. A. Sosa, A. Q. Terry, F. M. Kaldas, Y.-P. Jin, M. Rossetti, T. Ito, F. Li, R. S. Ahn, B. V. Naini, V. M. Groysberg, Y. Zheng, A. Aziz, J. Nevarez-Mejia, A. Zarrinpar, R. W. Busuttil, D. W. Gjertson, J. W. Kupiec-Weglinski, E. F. Reed, Disulfide high-mobility group box 1 drives ischemia-reperfusion injury in human liver transplantation. *Hepatology* **73**, 1158–1175 (2021).

56. A. Q. Terry, H. Kojima, R. A. Sosa, F. M. Kaldas, J. L. Chin, Y. Zheng, B. V. Naini, D. Noguchi, J. Nevarez-Mejia, Y.-P. Jin, R. W. Busuttil, A. S. Meyer, D. W. Gjertson, J. W. Kupiec-Weglinski, E. F. Reed, Disulfide-HMGB1 signals through TLR4 and TLR9 to induce inflammatory macrophages capable of innate-adaptive crosstalk in human liver transplantation. *Am. J. Transplant.* **23**, 1858–1871 (2023).

57. Q. Zhang, M. Raoof, Y. Chen, Y. Sumi, T. Sursal, W. Junger, K. Brohi, K. Itagaki, C. J. Hauser, Circulating mitochondrial DAMPs cause inflammatory responses to injury. *Nature* **464**, 104–107 (2010).

58. L. P. Westhaver, S. Nersesian, R. J. Arseneau, J. Hefler, B. K. V. Hargreaves, A. Edgar, Y. Azizieh, N. Cuesta-Gomez, D. L. Izquierdo, A. M. J. Shapiro, B. L. Gala-Lopez, J. E. Boudreau, Mitochondrial DNA levels in perfusate and bile during ex vivo normothermic machine correspond with donor liver quality. *Heliyon* **10**, e27122 (2024).

59. O. Yoshino, B. K. L. Wong, D. R. A. Cox, E. Lee, G. Hepworth, C. Christophi, R. Jones, A. Dobrovic, V. Muralidharan, M. V. Perini, Elevated levels of circulating mitochondrial DNA predict early allograft dysfunction in patients following liver transplantation. *J. Gastroenterol. Hepatol.* **36**, 3500–3507 (2021).

60. J. Pollara, R. W. Edwards, L. Lin, V. A. Bendersky, T. V. Brennan, Circulating mitochondria in deceased organ donors are associated with immune activation and early allograft dysfunction. *JCI Insight* **3**, e121622 121622 (2018).

61. Y. Zhu, F. Xiao, Y. Wang, Y. Wang, J. Li, D. Zhong, Z. Huang, M. Yu, Z. Wang, J. Barbara, C. Plunkett, M. Zeng, Y. Song, T. Tan, R. Zhang, K. Xu, Z. Wang, C. Cai, X. Guan, S. Hammack, L. Zhang, Z. Shi, F.-L. Xiang, F. Shao, J. Xu, NINJ1 regulates plasma membrane fragility under mechanical strain. *Nature* **644**, 1088–1096 (2025).

62. T. Araki, D. B. Zimonjic, N. C. Popescu, J. Milbrandt, Mechanism of homophilic binding mediated by ninjurin, a novel widely expressed adhesion molecule. *J. Biol. Chem.* **272**, 21373–21380 (1997).

63. P. Dutkowski, M. Linecker, M. L. DeOliveira, B. Müllhaupt, P.-A. Clavien, Challenges to liver transplantation and strategies to improve outcomes. *Gastroenterology* **148**, 307–323 (2015).

64. T. Ito, B. V. Naini, D. Markovic, A. Aziz, S. Younan, M. Lu, H. Hirao, K. Kadono, H. Kojima, J. DiNorcia III, V. G. Agopian, H. Yersiz, D. G. Farmer, R. W. Busuttil, J. W. Kupiec-Weglinski, F. M. Kaldas, Ischemia-reperfusion injury and its relationship with early allograft dysfunction in liver transplant patients. *Am. J. Transplant.* **21**, 614–625 (2021).

65. Z. Czigany, I. Lurje, R. H. Tolba, U. P. Neumann, F. Tacke, G. Lurje, Machine perfusion for liver transplantation in the era of marginal organs-New kids on the block. *Liver Int.* **39**, 228–249 (2019).

66. N. Goldaracena, J. M. Cullen, D.-S. Kim, B. Ekser, K. J. Halazun, Expanding the donor pool for liver transplantation with marginal donors. *Int. J. Surg.* **82S**, 30–35 (2020).

67. V. G. Agopian, M. P. Harlander-Locke, D. Markovic, W. Dumronggittigule, V. Xia, F. M. Kaldas, A. Zarrinpar, H. Yersiz, D. G. Farmer, J. R. Hiatt, R. W. Busuttil, Evaluation of early allograft function using the liver graft assessment following transplantation risk score model. *JAMA Surg.* **153**, 436–444 (2018).

68. N. Selzner, M. Selzner, W. Jochum, P.-A. Clavien, Ischemic preconditioning protects the steatotic mouse liver against reperfusion injury: an ATP dependent mechanism. *J. Hepatol.* **39**, 55–61 (2003).

69. N. Percie Du Sert, A. Ahluwalia, S. Alam, M. T. Avey, M. Baker, W. J. Browne, A. Clark, I. C. Cuthill, U. Dirnagl, M. Emerson, P. Garner, S. T. Holgate, D. W. Howells, V. Hurst, N. A. Karp, S. E. Lazic, K. Lidster, C. J. MacCallum, M. Macleod, E. J. Pearl, O. H. Petersen, F. Rawle, P. Reynolds, K. Rooney, E. S. Sena, S. D. Silberberg, T. Steckler, H. Würbel, Reporting animal research: Explanation and elaboration for the ARRIVE guidelines 2.0. *PLoS Biol.* **18**, e3000411 (2020).

70. M. Charni-Natan, I. Goldstein, Protocol for primary mouse hepatocyte isolation. *STAR Protocols* **1**, 100086 (2020).

71. I. Wittig, H.-P. Braun, H. Schägger, Blue native PAGE. *Nat. Protoc.* **1**, 418–428 (2006).

72. O. Yakubovsky, K. Bahar Halpern, S. Shir, R. Novoselsky, A. Afriat, A. Egozi, T. Barkai, Y. Harnik, R. Hoefflin, Y. Korem Kohanim, O. Golani, I. Goliand, Y. Addadi, M. Kedmi, H. Keren-Shaul, L. Fellus-Alyagor, L. Prichislov, D. Hirsch, C. Mayer, R. Pery, N. Pencovich, T. Taner, I. Nachmany, S. Itzkovitz, A spatial atlas of the healthy human liver from live donors. *Nature* **653**, 1148–1157 (2026).
